# Polymerization of Aniline Derivatives to Yield Poly[*N*,*N*-(phenylamino)disulfides] as Polymeric Auxochromes

**DOI:** 10.1021/acs.macromol.1c01548

**Published:** 2021-11-12

**Authors:** James P. Grace, Evan S. Flitz, Dae Sun Hwang, Ned B. Bowden

**Affiliations:** Department of Chemistry, University of Iowa, Iowa City, Iowa 52242, United States

## Abstract

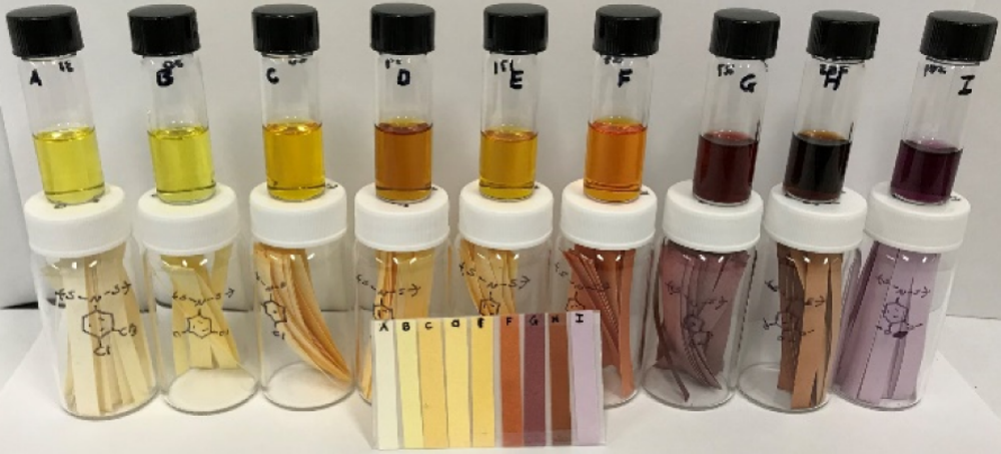

Polymerizations of phenylamines with a disulfide transfer reagent to yield poly[*N*,*N*-(phenylamino) disulfides] (**poly-NADs**) were investigated due to their unique repeat units that resulted in conjugation along the backbone that was perturbed by the aromatic rings and gave different colors for the polymers. These polymers were synthesized from 10 different anilines and sulfur monochloride in a step-growth polymerization. The polymers were characterized by nuclear magnetic resonance spectroscopy, size exclusion chromatography-multiangle light scattering, and UV–vis spectroscopy. These polymers possessed a polymeric backbone solely consisting of nitrogen and sulfur [−N(R)SS−], which was conjugated and yielded polymers of moderate molecular weight. Most notably, these polymers were an array of colors ranging from pale yellow to a deep purple depending on the substitution of the aromatic ring. The more electron-poor systems produced lighter yellow polymers, while the electron-rich systems gave orange, green, red, and even purple polymers.

## 1 Introduction

Enhancing and augmenting polymeric materials with sulfur atoms is a common practice in the production of materials with different morphologies and properties.^1−3^ Traditionally, this reaction has been done by processes such as vulcanization, to strengthen materials using elemental sulfur.^4−13^ Alternative methods have been developed for more versatile and precise ways to introduce sulfur widely into materials for a variety of applications. The discovery by Pyun et al. in 2013 that elemental sulfur can be polymerized with vinylic monomers accelerated research in sulfur-rich polymers to develop new polymerization methods and applications of these polymers.^14^ Sulfur-rich polymers often contain disulfide bonds that introduce inherent flexibility, a large electron-accepting capacity, and a weak (−S–S−) bond, which can be triggered in reversible chemical and redox reactions.^1,2,5,6,15−24^ These polymers are being investigated for applications as supramolecular linkages,^19,25^ encapsulation-controlled drug release,^21,26,27^ components for metal-ion batteries,^15,28−34^ and self-healing materials.^16,18^ Research into the synthesis and applications of heterosubstituted disulfide polymers will expand the properties of sulfur-rich polymers by the integration of novel atoms bonded to sulfur, but they are less well studied.^35,36^ Polymers based on bonds between sulfur and nitrogen are greatly understudied in macromolecular science despite the recent interest in polysulfides due to their unique physical and chemical properties. Early work in this field has demonstrated that polymers based on bonds between N and S can be synthesized in high yields with the S atoms as divalent to hexavalent.^22,27,35−38^ These polymers can take advantage of the polarized N–S bonds, while allowing conjugation through the NS framework.^39−41^ Polymers based on bonds between N and S have found applications in medicine,^26,27,42,43^ energy storage,^35,36,42,44^ flame retardants,^45^ and metal-ion detection.^46^

**Figure 1:**
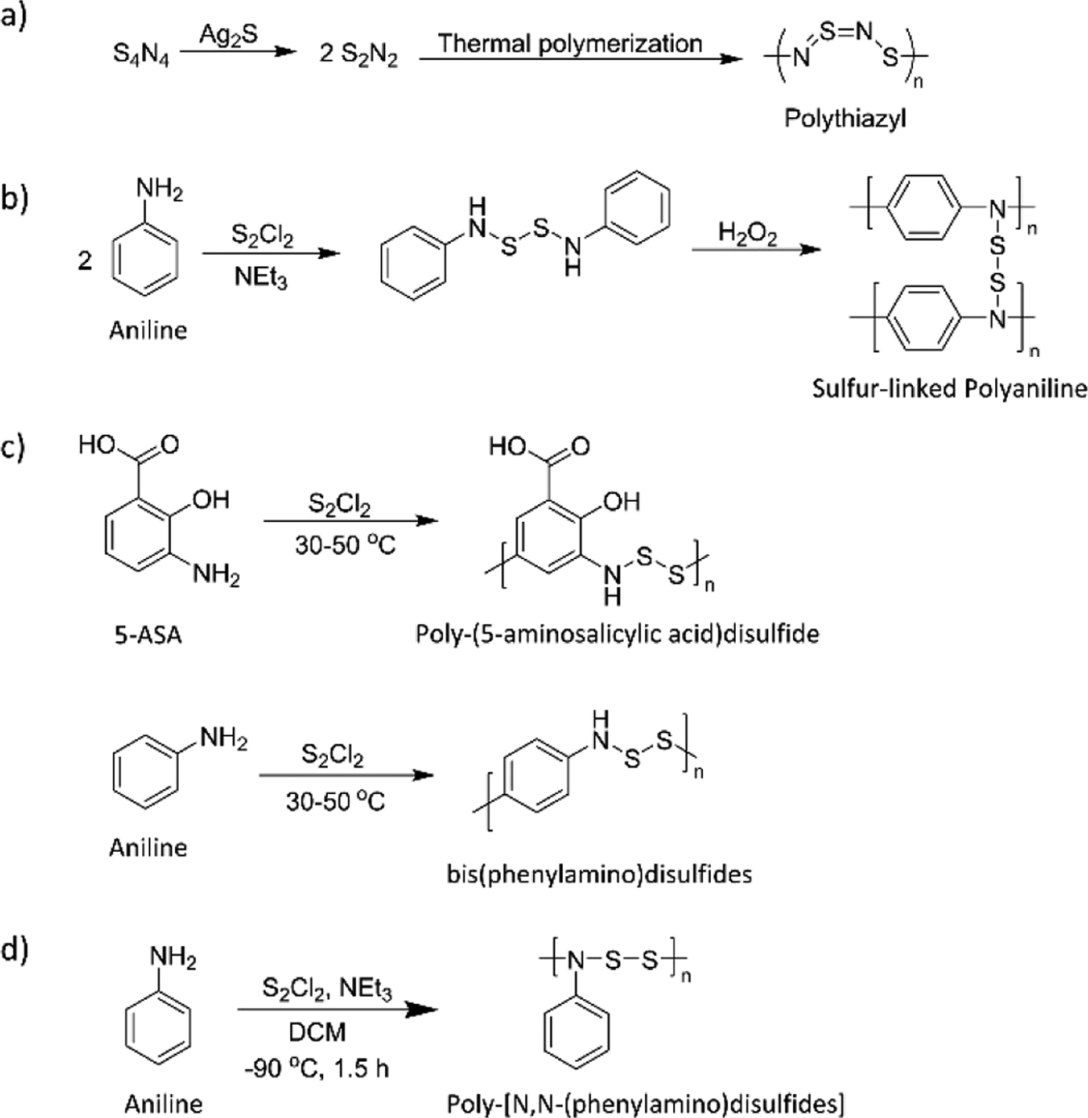
(a) Structure and reaction conditions for the synthesis of polythiazyl is shown. (b) Sulfur cross-linked polyaniline was synthesized by the initial synthesis of bis(*N*,*N*′-aniline disulfide) followed by chemical polymerization with H_2_O_2_. (c) Polymerization conditions shown were reported to yield poly-(5-aminosalicylic acid)disulfide and poly-bis(phenylamino)disulfide. (d) Synthesis of poly[*N*,*N*-(phenylamino)disulfides] from prior work by us is shown.

An important example of a polymer based on N–S bonds is poly(sulfur nitride), which is also known as polythiazyl, that was first characterized in 1953 (Figure 1a).^47−52^ This polymer is composed entirely of alternating N and S atoms and is referred to as a metallic polymer due to its high electrical conductivity. A limitation of polythiazyl is its simplicity, its structure does not lend the polymer to systematic variation because it is only composed of alternating S and N atoms. Other conjugated polymers based on N–S bonds were synthesized, such as in a 2004 report, where the monomer containing the NSSN functional group was electrically or chemically polymerized to yield a blue-green cross-linked polymer (Figure 1b).^36^ This polymer was investigated for its properties as an electrode material and found to have a high power density due to the presence of the S–S bonds. Due to its highly cross-linked structure, it was not fully characterized. In related works, conjugated polymers based on the aminodisulfide functional group were reported (Figure 1c).^53,54^ This polymer was synthesized by a reaction between 5-aminosalicyclic and sulfur monochloride (S_2_Cl_2_). It was reported in 1998 and characterized by UV–vis spectroscopy. The polymerization was proposed to proceed by the reaction of the amine and sulfur monochloride (S_2_Cl_2_) followed by electrophilic aromatic substitution to yield the C–S bond in the product. Similarly, the polymerization of aniline with S_2_Cl_2_ was reported in 1974 by different authors to proceed by similar mechanisms to yield polymers with the NSS functional group.^55^ This polymer was thermally stable and was found to be paramagnetic. Unfortunately, neither of these two polymers were fully characterized to prove their structures.

In prior works by us, aniline was polymerized with sulfur monochloride to yield a different polymer than that reported in the literature (Figure 1d).^37^ We completed our polymerizations by reacting aniline and S_2_Cl_2_ at −78 °C in the presence of triethylamine. This is in contrast to the synthesis reported in Figure 1c, where S_2_Cl_2_ and aniline were heated in the absence of a base to 50 °C to yield a polymer. The polymer that we synthesized was readily characterized by NMR spectroscopy and SEC that described a polymer of molecular weight of 4300 g mol^–1^.

We hypothesized that the polymerization of phenylamines with sulfur monochloride would yield conjugated polymers with the general structure shown in Figure 1d. Based on prior works that demonstrated conjugation through N–S bonds, we further hypothesized that the colors of these polymers could be varied by the substituents on the aromatic rings. In this article, we report the synthesis of these polymers and characterization of their colors from bright yellow to deep purple and green. We also investigated if the polymerization proceeded through the nitrogen or a combination of the amine and aromatic rings as proposed by others. Additionally, preliminary experiments were conducted to probe their sensitivity to 2-mercaptoethanol (2-ME) for applications as colorimetric sensors.

## 2 Experimental Section

### 2.1 Materials

The phenylamines were purchased from Sigma-Aldrich and purified by sublimation or distillation prior to use. Triethylamine (TEA) was purchased from Fischer Scientific and used without further purification. Sulfur monochloride was purchased from Sigma-Aldrich and distilled over crystalline elemental sulfur within a week of use and stored at 0 °C under nitrogen. Hydrogen and nitrogen gases were purchased from PraxAir. DCM and THF were purchased from Sigma-Aldrich and dried over anhydrous MgSO_4_, distilled, deoxygenated by the freeze–pump-thaw cycle repeated in triplicate, and stored over molecular sieves under a nitrogen atmosphere. ^1^H and ^13^C NMR spectra were recorded on AVANCE 400 and 100 MHz NMR instruments, respectively. Column chromatography was performed using a SilicaFlash F60 silica gel (230–400 Mesh). HRMS was conducted on a Waters Q-Tof Premier.

### 2.2 General Procedure for the Synthesis of Poly-NADs

All poly[*N*,*N*-(phenylamino)disulfides] (**A**–**I**, **L**, and **M**) and poly[*N*,*N*-(cyclohexylamino)disulfide] (**J**) were synthesized by the same general procedure. S_2_Cl_2_ (10 mmol, 0.8 mL) was added to a flame-dried 100 mL Schlenk flask with a stir bar and dissolved in dry/deoxygenated DCM (10 mL). The flask was equipped with a pressure equalizing addition funnel and charged with an appropriate phenylamine monomer (10 mmol) and TEA (22 mmol, 2.2 equiv) dissolved in dry/deoxygenated DCM (10 mL). The system was purged with N_2_ gas prior to the reaction and cooled to −90 °C in an acetone/N_2(l)_ bath for 30 min. The amine solution was added dropwise to the cooled S_2_Cl_2_ solution for approximately 20–30 min (0.5 drop/sec). The reaction was allowed to stir for an additional 30 min at −90 °C before being removed from the bath and warming to room temperature for 30 min. The reaction mixture was concentrated by half to aid in precipitation. Unless otherwise stated, polymers were purified by precipitation from minimal DCM into a stirred solution of cold (0 °C) MeOH (40 mL) followed by vacuum filtration. This procedure was repeated in triplicate and dried under reduced pressure (100–600 mTorr) overnight to obtain a clean polymer. ^1^H NMR characterization of poly-NADs was performed with a Bruker AVANCE 400 MHz at room temperature.

Poly[*N*,*N*-(4-chloro-3-trifluoromethyl phenylamine)disulfide] (**A**); isolated pale yellow powder (0.26 g, 10%). ^1^H NMR (400 MHz, CDCl_3_): δ 7.9–7.0 (broad, m, 3H).

Poly[*N*,*N*-(3,5-dichlorophenylamine)disulfide] (**B**); isolated pale yellow powder (0.42 g, 19%). ^1^H NMR (400 MHz, CDCl_3_): δ 7.5–6.8 (broad, m, 3H).

Poly[*N*,*N*-(4-chlorophenylamine)disulfide] (**C**); isolated yellow powder (0.58 g, 31%). ^1^H NMR (400 MHz, CDCl_3_): δ 7.8–7.0 (broad, m, 4H).

Poly[*N*,*N*-(phenylamine)disulfide] (**D**); isolated yellow powder (0.27 g, 21%). ^1^H NMR (400 MHz, CDCl_3_): δ 7.6–6.7 (broad, m, 5H).

Poly[*N*,*N*-(2,4,6-trimethylaniline)disulfide] (**E**); isolated yellow powder (0.15 g, 15%). ^1^H NMR (400 MHz, CDCl_3_): δ 6.9–6.4 (broad, m, 2H), δ 2.4–2.0 (broad, m, 9H).

Poly[*N*,*N*-(4-methylphenylamine)disulfide] (**F**); isolated orange powder (0.37 g, 23%). ^1^H NMR (400 MHz, CDCl_3_): δ 7.6–6.9 (broad, m, 4H), δ 2.4–2.2 (broad, m, 3H).

Poly[*N*,*N*-(3,4-methylenedioxyphenylamine)disulfide] (**G**); isolated purple powder (0.47 g, 24%). ^1^H NMR (400 MHz, CDCl_3_): δ 7.2–6.5 (broad, m, 3H), δ 6.1–5.8 (broad, m, 2H).

Poly[*N*,*N*-(cyclohexylamine)disulfide] (**J**); isolated ivory white powder (0.30 g, 19%). ^1^H NMR (400 MHz, CDCl_3_): δ 3.1–3.6 (broad, m, 1H), δ 2.2–1.9 (broad, m, 4H), 1.6–1.0 (broad, m, 6H).

Poly[*N*,*N*-(2-aminoanthracene)disulfide] (**M**); isolated dark green powder (0.23 g, 27%). ^1^H NMR (400 MHz, CDCl_3_): δ 7.0–7.5 (broad, m, 9H).

### 2.3 Synthesis of Pentamethylnitrobenzene

A modified literature procedure was used to nitrate pentamethylbenzene.^56^ Pentamethylbenzene (4.7 g, 31.6 mmol) was dissolved in stirring acetonitrile (40 mL). Silver nitrate (5.4 g, 31.6 mmol) was added all at once and the reaction mixture was cooled to 0 °C in an ice bath for 15 min. A solution of saturated boron trifluoride in acetonitrile (25 mL, 15% solution) was added to the reaction mixture in a slow stream to maintain 0 °C. The reaction mixture was then removed from the ice bath and warmed to room temperature. The reaction mixture was allowed to stir for 15 h at r.t. The reaction mixture was quenched with ice water (70 mL) and extracted diethyl ether (2 × 50 mL). The combined organic layers were washed with a sequence of water (50 mL), saturated sodium bicarbonate (50 mL), water (2 × 50 mL), and brine solution (50 mL). The organic phase was then dried over anhydrous MgSO_4_ and concentrated under reduced pressure yielding a crude orange solid. This was recrystallized from boiling EtOH to yield pentamethyl nitrobenzene as a vibrant yellow solid (4.82 g, 77%). ^1^H NMR (400 MHz, CDCl_3_): δ 2.24 (s, 3H), δ 2.22 (s, 6H), δ 2.15 (s, 6H).

### 2.4 Synthesis of Pentamethylaniline

Pure pentamethylnitrobenzene (**PNMB**) (0.72 g, 3.7 mmol) was dissolved in dry THF (20 mL) and placed in a 100 mL, Teflon lined parr reactor with a stir bar. A palladium catalyst (0.15 g, 10% Pd/C) was added and the parr reactor was assembled. The reaction vessel was then filled to 500 psi and vented three times with H_2_ gas before being filled a final time with H_2_ to a final pressure of 1000 psi. This was allowed to stir at room temperature for 48 h. The crude solution was filtered through celite, concentrated under reduced pressure, and purified by column chromatography (silica gel, 8.5:1.5 hexanes/ethyl acetate), yielding pentamethyl aniline as a white crystalline solid (0.11 g, 18%). ^1^H NMR (400 MHz, CDCl_3_): δ 3.48 (broad s, 2H), δ 2.21 (s, 6H), δ 2.18 (s, 3H), δ 2.12 (s, 6H). ^13^C NMR (100 MHz, CDCl_3_): δ 140.3, 132.4, 125.1, 118.2, 16.8, 16.5, 13.9. HRMS: (M + H)^+^ C_11_H_17_N calcd, 164.1439; found, 164.1434.

### 2.5 Polymerization to Yield Poly[*N*,*N*-(pentamethylaminobenzene)disulfide] (**L**)

S_2_Cl_2_ (0.67 mmol, 0.054 mL) was added to a flame-dried 25 mL round-bottomed flask under N_2_ with a stir bar and dissolved in dry/deoxygenated DCM (2.0 mL, 1 mM). The flask was sealed and flushed with N_2_ gently before cooling to −90 °C in an acetone/N_2(l)_ bath. Pentamethylaniline (0.67 mmol, 0.11 g) and triethylamine (1.47 mmol, 0.21 mL, 2.2 equiv) were dissolved in dry/deoxygenated DCM (2.0 mL). The amine solution was added dropwise to the cooled S_2_Cl_2_ solution via a syringe for approximately 12 min. The reaction mixture was allowed to stir for an additional 30 min at −90 °C before being removed from the bath and warming to room temperature for 15 min. The reaction mixture was concentrated by half and precipitated into a stirred solution of cold (0 °C) MeOH (20 mL) followed by vacuum filtration. This procedure was repeated in duplicate and dried under reduced pressure (100–600 mTorr) overnight to obtain poly[*N*,*N*-(pentamethylaminobenzene)disulfide] as a pale yellow powder (0.08 g, 53%). ^1^H NMR (400 MHz, CDCl_3_): δ 2.35 (broad, s, 3H), δ 2.30 (broad, s, 6H), 2.21 (broad, s, 6H).

### 2.6 Modified Isolation Procedure for Polymers Soluble in MeOH

The crude reaction was concentrated under reduced pressure. The polymer was precipitated into a stirred solution of hexanes (30 mL), redissolved in a minimal volume of acetone, and filtered through celite to remove excess insoluble amine salts. The polymer was reprecipitated into water followed by vacuum filtration and the removal of the residual solvent under vacuum to yield polymers **H** and **I**.

Poly[*N*,*N*-(3,4,5-trimethoxyphenylamine)disulfide] (**H**); isolated dark red powder (0.40 g, 16%). ^1^H NMR (400 MHz, CDCl_3_): δ 7.0–6.0 (broad, m, 2H), δ 4.0–3.5 (broad, m, 9H).

Poly[*N*,*N*-(3,4-dimethoxyphenylamine)disulfide] (**I**); isolated dark purple powder (0.25 g, 10%). ^1^H NMR (400 MHz, CDCl_3_): δ 7.9–7.1 (broad, m, 3H), δ 4.3–3.2 (broad, m, 6H).

### 2.7 Bis-(*N*-methylaniline disulfide) (**K**)

This chemical was synthesized using a similar method as the polymers (**A**–**G**). S_2_Cl_2_ (5 mmol, 0.4 mL) was added to a flame-dried 100 mL Schlenk flask with a stir bar and dissolved in dry/deoxygenated DCM (10 mL, 0.5 mM). The flask was equipped with a pressure equalizing addition funnel and charged with freshly distilled *N*-methylaniline (10 mmol) and TEA (22 mmol, 2.2 equiv) dissolved in dry/deoxygenated DCM (10 mL). The system was purged with N_2_ gas prior to the reaction and cooled to −90 °C in an acetone/N_2(l)_ bath for 30 min. The amine solution was added dropwise to the cooled S_2_Cl_2_ solution for approximately 20–30 min (0.5 drop/sec). The reaction mixture was allowed to stir for an additional 30 min at −90 °C before being removed from the bath and warming to room temperature for 30 min. The reaction mixture was concentrated under reduced pressure to yield an orange solid (^1^H NMR yield = 84%). The crude product was purified by an acetone wash to remove excess triethylamine salts followed by column chromatography (hexanes/EtOAc 7:3). A yellow crystalline solid (0.70 g) was isolated. ^1^H NMR (400 MHz, CDCl_3_): δ 7.20 (dt, 2H), δ 7.02 (dt, 2H) δ 6.85 (d, 1H) δ 2.99 (s, 3H). ^13^C NMR (100 MHz, CDCl_3_) δ 149.53, 128.75, 120.67, 117.48, 43.98. HRMS (M + H)^+^ C_14_H_17_N_2_S_2_ calcd, 277.0833; found, 287.0827.

### 2.8 Thermogravimetric Analysis

Thermogravimetric analysis (TGA) was performed on a TA instruments’ TGA Q500 under a N_2_ atmosphere. An aluminum pan was used and a ramp rate of 5 °C/min equilibrated at 500 °C was used.

### 2.9 Elemental Analysis

Elemental analysis was conducted on a Thermo FlashSmart 2000 equipped with a quartz reduction tube with copper oxide and copper wires. The combustion was conducted at 950 °C.

### 2.10 SEC-MALS Determination of MW of Poly-NADs

Size-exclusion chromatography (SEC) was performed on a Waters Styragel Column (7.8 mm × 300 mm, 500 Da to 30 KDa) using a Waters 515 HPLC pump (0.5 mL/min) equipped with an auto-injection port (50 μL inj. vol). All polymers were measured using a Wyatt Dawn Heleos II (664 nm) multi-angle light scattering detector in line with a Wyatt T-rEX refractometer. The system was calibrated in HPLC toluene and normalized using polystyrene standards in HPLC THF. Samples were weighed out using a Radwag Microbalance MYA 21.3Y, dissolved in HPLC THF, and filtered prior to injection with Tisch Scientific 0.1 μm PTFE syringe filters. Molecular weights (MWs) were calculated from the estimated d*n*/d*c* values given by assuming 100% mass recovery.

### 2.11 Ultraviolet–Visible Characterization of Poly-NADs

Ultraviolet–visible spectra of all polymers were collected on an Agilent Cary 5000 UV–Vis/NIR spectrophotometer system and software using a double-front method compared against pure HPLC-chloroform (250–800 nm). All samples were dissolved in HPLC-chloroform and measured in Hëllma analytics high precision cells made from optical glass (10 mm pathlength). All samples were prepared at a starting concentration between 30 and 50 mM in 1 mL of HPLC CHCl_3_ and serial diluted by half for each subsequent sample. All data were processed using Microsoft Excel for UV–vis plots and calculations and Fityk peak fitting software (gaussian, Lev-Mar method). Molar absorptivities were calculated using Beer’s Law and averaging the results of five peak fit absorption spectra at varying concentrations. Additional, relevant graphical data and calculations can be found in the Supporting Information.

### 2.12 Thiol Sensitivity Qualitative Determination

All polymer samples were prepared at a ∼30 mM concentration using dry dichloromethane. After initial imaging, 2-mercaptoethanol (0.5 mL, 50 equiv) was added and mixed thoroughly. Subsequent images were taken at 2 h, 5 h, and 24 h after addition.

### 2.13 Cross-Linker Studies of Poly-NADs with *p*-Phenylenediamine

S_2_Cl_2_ (11 mmol, 0.88 mL) was added to a flame-dried 100 mL Schlenk flask with a stir bar and dissolved in dry/deoxygenated DCM (10.0 mL). The flask was equipped with a pressure equalizing addition funnel, charged with the appropriate phenylamine monomer (10 mmol), TEA (24 mmol, 2.2 equiv. to NH_2_), and cross-linking agent *p*-phenylenediamine (ppda) (0.05 equiv), and dissolved in dry/deoxygenated DCM (10 mL). The system was purged with N_2_ gas prior to the reaction and cooled to −90 °C in an acetone/N_2(l)_ bath for 30 min. The amine solution was added dropwise to the cooled S_2_Cl_2_ solution for approximately 20–30 min (0.5 drop/sec). The reaction mixture was allowed to stir for an additional 30 min at −90 °C before being removed from the bath and warming to room temperature for 30 min. The reaction mixture was concentrated by half to aid in precipitation. Polymers were purified by precipitation from minimal DCM into a stirred solution of cold (0 °C) MeOH (60 mL) followed by vacuum filtration. This procedure was repeated in triplicate and dried under reduced pressure (100–600 mTorr) overnight to obtain a clean polymer (D-ppda = 1.47 g, 94%: F-PPDA = 1.35 g, 80%). MWs were determined by SEC-MALS analysis.

## 3 Results and Discussion

### 3.1 Synthesis of the Polymers

**Figure 2:**
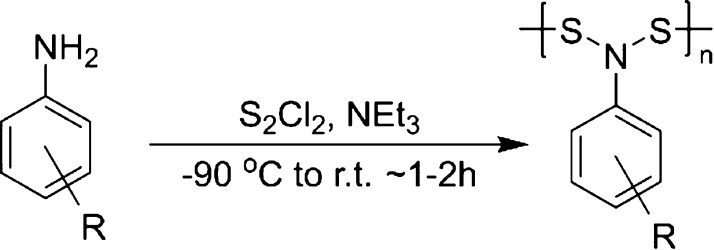
Reaction for synthesis of poly-NADs.

**Figure 3:**
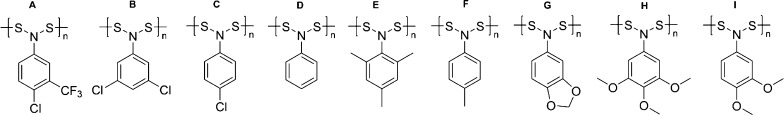
Polymers that were synthesized are shown in order of their λ_max_ in the visible region.

The poly[*N*,*N*-(phenylamine)disulfides] were synthesized by a condensation polymerization reaction of a phenylamine with S_2_Cl_2_ in the presence of triethylamine in anhydrous DCM at reduced temperatures (−90 °C) (Figure 2). At room temperature, the reaction was highly exothermic and rapid, yielding little to no desired polymerization due to competing side reactions. S_2_Cl_2_ was cooled in DCM to −90 °C, and a phenylamine monomer was slowly added over 20 min. After the aniline was added, the reaction was allowed to warm to room temperature with stirring for an additional 60 min. The polymers were then isolated as described in the Experimental Section. This procedure was followed for nine phenylamines as well as cyclohexylamine (Figure 3). The phenylamines possessed a variety of electron-withdrawing or electron-donating substituents that allowed their effect on color to be investigated.

The polymerizations were completed under N_2_, but the polymers were handled under atmospheric conditions. As solids, the polymers were found to be air and moisture stable for months without any evidence of degradation.

### 3.2 Investigation of Alternative Disulfide Transfer Reagents for Polymerization

**Figure 4:**
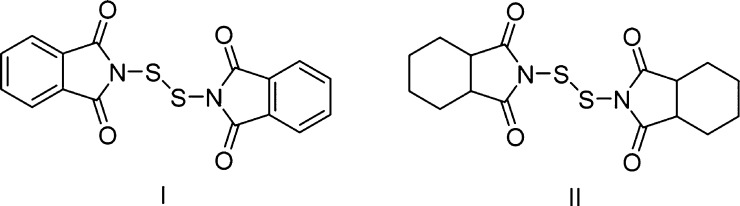
Disulfide transfer reagents bis-phthalimide disulfide (**I**) and bis-2,3,4,5-tetrahydrophthalimide disulfide (**II**).

S_2_Cl_2_ is a ubiquitous disulfide transfer reagent in organic chemistry; however, it suffers from high reactivity leading to potential side reactions if not controlled carefully. Therefore, more mild disulfide transfer reagents were investigated to determine if they could be used in the polymerization of the aniline derivatives. The efficacy of polymerization with all aniline derivatives were tested using both bis-phthalimide disulfide (I) as well as bis-2,3,4,5-tetrahydrophthalimide disulfide (II) (Figure 4). These reactions were repeated at a variety of temperatures, times, and in the presence and absence of bases (NEt_3_ or tribenzylamine). The reactions were sluggish and only low conversions were observed by ^1^H NMR spectroscopy and no polymer was observed as confirmed by GPC.

### 3.3 Characterization of the MW and Compositions of the Polymers

**Figure 5:**
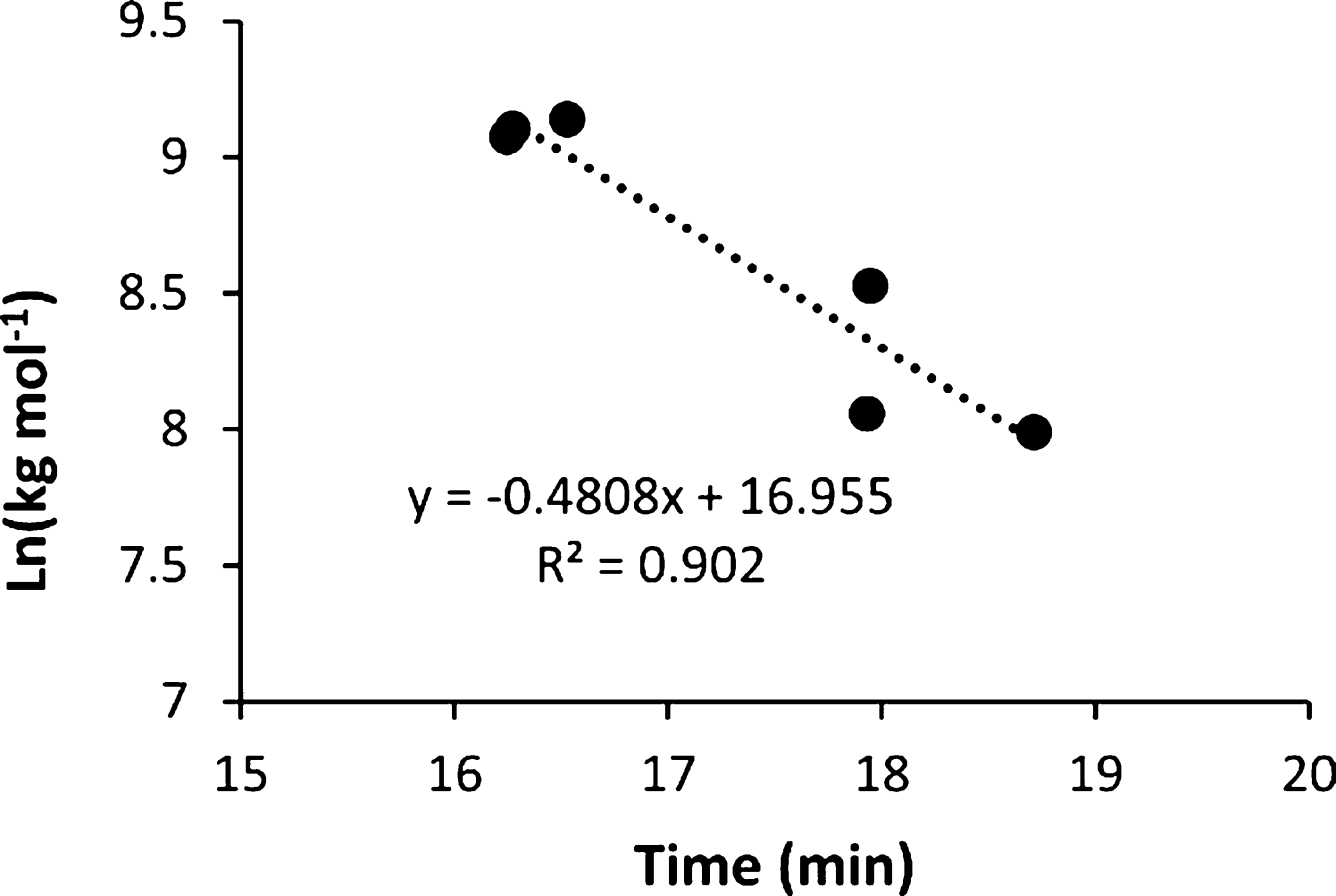
Refractive index (RI) retention time calibration curve generated from known MW of poly-NADs.

The polymers were characterized by SEC-MALS to find their absolute MW and polydispersities (Table 1). For three polymers (**G**, **H**, and **I**), the wavelength used by a MALS detector (664 nm) was partially absorbed by the polymer. This absorption resulted in negative light scattering signals, which made an accurate determination of MW impossible. A calibration curve was generated to measure the approximate MW of the polymer from the retention time of the dRI signal. Initially, a calibration curve was generated with polystyrene standards; however, application of this curve resulted in very low and even negative, MWs for the polymers. Therefore, a calibration curve was generated using the poly[*N*,*N*-(phenylamine)disulfide]s that had their MW measured using dRI and light scattering (Figure 5). This curve provided reasonable MW and polydispersities for polymers **G**, **H**, and **I**. These polymers possessed a number of repeat units that ranged from a low of 12 for **G** and a high of 47 for **E**.

**Table 1:** MW and Dispersity (Đ) of Poly-NADs (**A–I**)

Poly-NAD	MW (g/mol)	dispersity (Đ)
A	8700	1.29
B	10,000	1.46
C	3000	1.21
D	4500	1.91
E	8900	1.84
F	3200	1.33
G	2300^a^	1.40
H	3500^a^	1.51
I	8200^a^	1.19

a Measured using a calibration curve of the MW of polymers **A** through **F**.

To confirm the structure of the polymers and the presence of sulfur, the elemental analyses of all representative poly-NADs (**A–I**) were measured using a CHNS probe (Table S2). The CHNS composition was measured two times for each polymer and the average values are shown. These results demonstrate that the polymers contained sulfur at levels that were close to the expected values. This result was important because the characterization of the polymers by NMR spectroscopy did not report the presence of sulfur, but it was inferred from the composition of the materials.

### 3.4 Investigation of Possible Side Reactions between the Aromatic Rings and S_2_Cl_2_

Although the amine in the phenylamines was the strongest nucleophile, the possibility that the aromatic ring reacted with S_2_Cl_2_ through electrophilic aromatic substitution was also considered and investigated by several methods. First, the polymerization of 2,4,6-trimethylaniline with S_2_Cl_2_ was investigated because this monomer had methyl groups at the ortho and para positions to the −NH_2_ on the ring that would prevent S_2_Cl_2_ from reacting with the ring at the preferred EAS positions. This monomer polymerized well and yielded a poly-NAD **E** with the second highest MW reported here.

**Figure 6:**
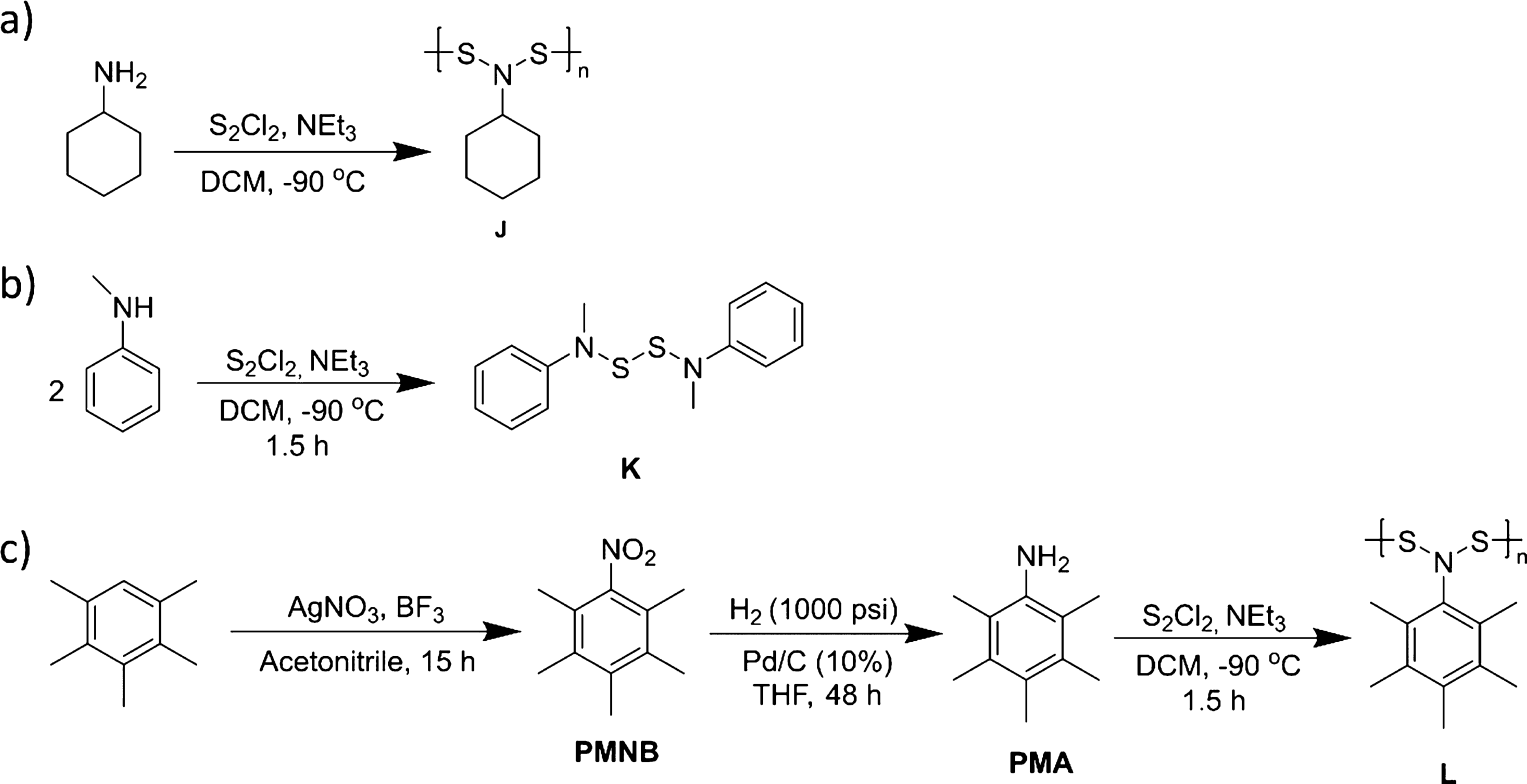
(a) Reaction of cyclohexylamine with S_2_Cl_2_ and base yielded **J**. (b) Reaction of *N*-methylaniline with S_2_Cl_2_ and base yielded **K**. (c) Sequence of reactions to afford pentamethylaniline (**PMA**) which was polymerized with S_2_Cl_2_ and base to yield **L**.

In a second method to investigate if a reaction solely between an amine and S_2_Cl_2_ would yield a polymer, cyclohexylamine was polymerized with S_2_Cl_2_ to yield poly[*N*,*N*-(cyclohexylamine)disulfide] (**J** in Figure 6a). This reaction was completed using the same procedure as for the phenylamines and resulted in a polymer with a Mw of 6000 g mol^–1^ with a PDI of 1.39. This result demonstrated that a primary amine without an aromatic ring could be polymerized solely through the amine to yield a polymer with a MW similar to those measured when phenylamines were reacted.

In a third method, we reacted *N*-methylaniline with S_2_Cl_2_ and triethylamine under the same reaction conditions as the polymerizations to yield bis-*N*-methylaniline disulfide (**K**), as shown in Figure 6b. The reaction yielded the desired product in a yield of 84% as measured by NMR spectroscopy. Importantly, no peaks in the ^1^H NMR spectrum that were consistent with the reaction of the aromatic ring with S_2_Cl_2_ were observed. This reaction demonstrates that the secondary position on the amine is reactive to S_2_Cl_2_ at low temperatures over the aromatic ring. This result supports the conclusion that the polymer is primarily formed through nitrogen and not by undesired aromatic substitution under these conditions.

In a final study, the polymerization of pentamethylaniline with S_2_Cl_2_ was completed to investigate if an aniline derivative that could not react through the aromatic ring would yield a polymer (Figure 6c). This polymerization was successful and yields a polymer with a value of MW of 3100 g mol^–1^ with a PDI of 1.25.

To investigate if a slight excess of S_2_Cl_2_ could yield cross-linked polymers with higher MW than those reported in Table 1, the polymerization of *p*-toluidine, 3,5-dichloroaniline, and 2-aminoanthracene was completed because they possessed open sites on the aromatic rings for EAS, and the polymers had very low solubility in MeOH and thus were easy to isolate. These monomers were reacted with a 1.1 or 1.2 equivalence of S_2_Cl_2_ and excess triethylamine at various conditions to promote EAS (Table 2). It was expected that if the aromatic ring was likely to undergo EAS, the extra equivalence of S_2_Cl_2_ would produce a cross-linked polymer with a significantly higher MW. If little to no EAS occurred, then the MW would be low due to the excess of one of the monomers in the step polymerization. These polymerizations were unsuccessful, and no polymers precipitated which further suggests that there is little to no cross-linking through the aromatic ring.

**Table 2:** Experiments with Increased Equivalence of S_2_Cl_2_

starting aniline	equivalence of S_2_Cl_2_^a^	temperature of addition	temperature of reaction	length of reaction	yield (%), MW
3,5-dichloroaniline	1.1	–90 °C	r.t.	1.5 h	no precipitate
2-aminoanthracene	1.1	–90 °C	r.t.	1.5 h	no precipitate
4-methylaniline	1.1	–90 °C	r.t.	1.5 h	no precipitate
4-methylaniline	1.1	–90 °C	r.t.	24 h	no precipitate
4-methylaniline	1.1	0 °C	r.t.	1.5 h	no precipitate
4-methylaniline	1.1	0 °C	r.t.	24 h	no precipitate
4-methylaniline	1.2	0 °C	r.t.	1.5 h	no precipitate
4-methylaniline	1.2	r.t.	40 °C	1.5 h	no precipitate

a All reactions used 2.2 equiv of NEt_3_ in relation to S_2_Cl_2_These experiments demonstrate that the polymerizations proceeded by reaction between the nitrogens and S_2_Cl_2_. Only very small amounts or none of the side reaction where the aromatic rings reacted with S_2_Cl_2_ are consistent with these experiments.

### 3.5 Cross-Linking Poly-NADs with an Aromatic Phenylenediamine and an Aliphatic Diamine

**Figure 7:**
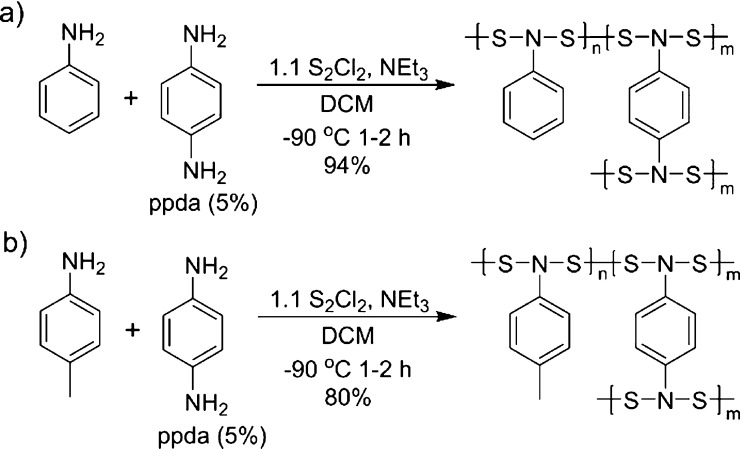
Polymerization reaction schemes are shown for the reaction of (a) **D** and (b) **F** with ppda to yield cross-linked polymers.

Two studies were done to demonstrate the possibility to improve the yield and MW of these polymers by adding in 5% cross-linking diamines while maintaining the 1:1 equivalence between amine and S_2_Cl_2._ The physical characteristics of the polymers were measured and compared with the non-cross-linked polymers (Figure 7 and Table 3).

**Table 3:** MW, Dispersity (*D̵*), and Isolated Yields of Polymers with and without ppda

polymer identification	equivalence of S_2_Cl_2_	equivalence of CL	equivalence of aniline	yield (%)	MW (g/mol)	dispersity (*D̵*)
poly-NAD **D**	1.0	N/A	1.0	21	4500	1.91
poly-NAD **F**	1.0	N/A	1.0	23	3200	1.33
poly-NAD **D-ppda**	1.1	0.05	1.0	94	15,200	1.69
poly-NAD **F-PPDA**	1.1	0.05	1.0	80	16,300	1.75

When the same polymerization procedures were completed with 5% of the cross-linker *p*-phenylenediamine (ppda), the observed molecular weight increased significantly (4500 g mol^–1^ for poly-NAD **D** to 15,200 g mol^–1^ for poly-NAD **D-PPDA**, and 3200 g mol^–1^ for poly-NAD **F** to 16,300 g mol^–1^ for poly-NAD **F-PPDA**) and allowed for improved isolation yields of 94% for poly-NAD **D-PDA** and 80% for poly-NAD **F-PPDA**. It was observed that these polymers had similar visible absorptions as the non-cross-linked analogues with the visible absorption only shifting from ∼10 nm (424 to 434 nm for poly-NAD **D**, and 429 to 439 nm for Poly-NAD **F**) (Figure S37). This hypsochromic shift was attributed to the increased electron donation from the diamine. These studies show that low concentrations of a cross-linker can increase the MW and isolated yield without large changes in the visible absorption.

### 3.6 Peak Fitting of UV–Vis Spectra to Extract Visible Absorptions

**Figure 8:**
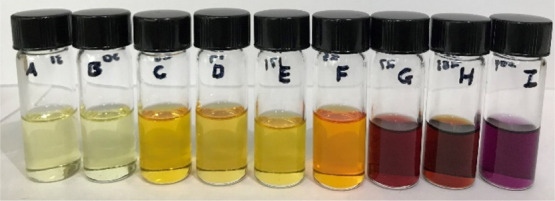
Solutions of poly-NADs (**A**–**I**) auxochromes at ∼2 mg/mL in DCM.

The poly-NADs possessed a wide range of colors which mostly followed the effects of the electron-donating and -withdrawing groups on the aromatic rings. More electron-withdrawing substituents led to a shorter absorption wavelength resulting in a yellow transmitted color, while the more electron-donating substituents on the rings lead to a longer wavelength and red shift in the visible absorbance. In the solution phase, this trend is quite apparent (Figure 8). The UV–vis spectra were investigated to characterize the adsorptions.

**Figure 9:**
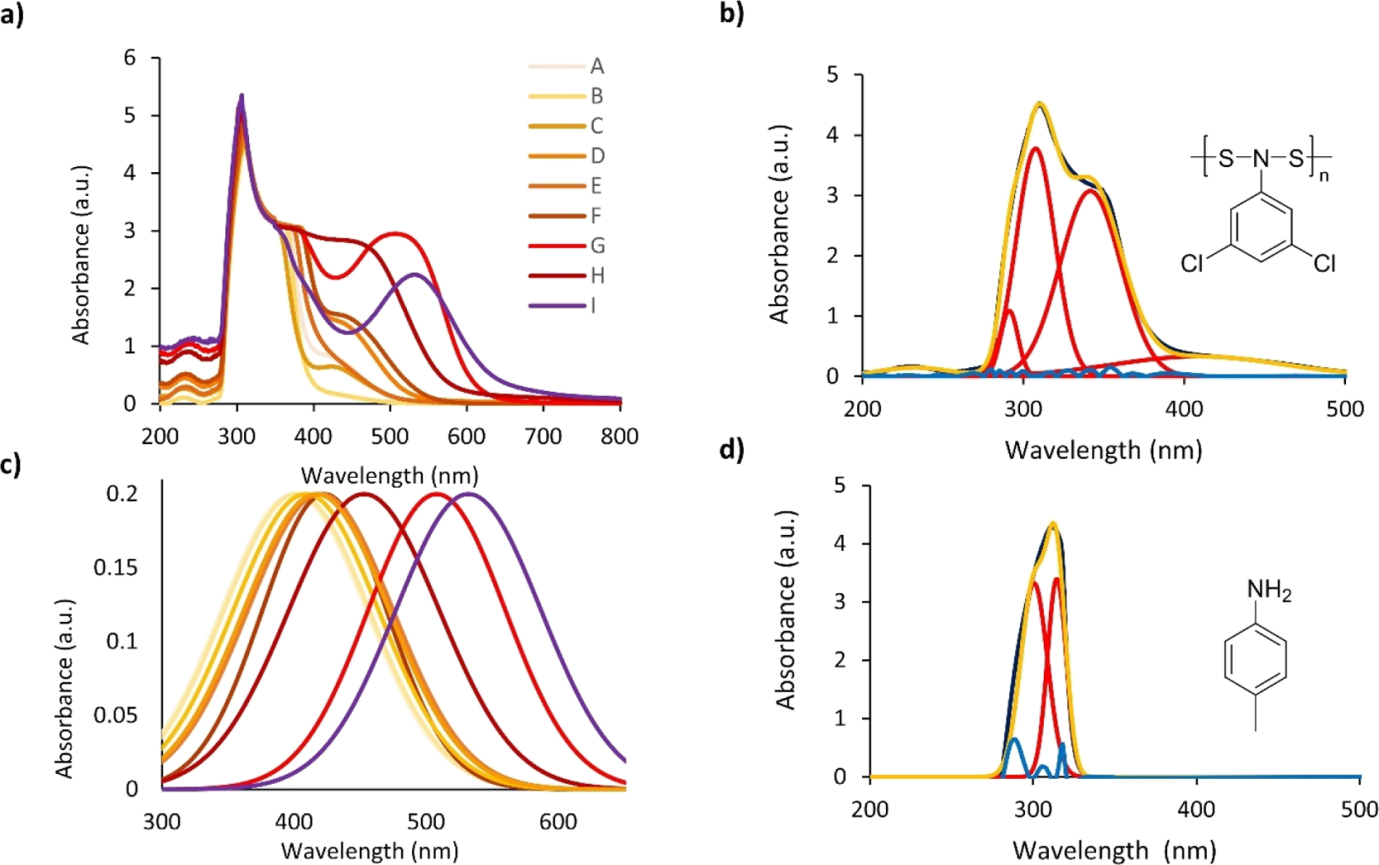
(a) Aggregate UV–vis graphs of all poly-NADs (**A**–**I**) overlapped at similar concentrations (∼5 mg/mL poly-NADs in CHCl_3_). (b) Example of the peak fit of poly[*N*,*N*-(3,5-dichlorophenylamine)disulfide] (**B**). Dark blue = UV–Vis measured spectrum, red = gaussian peak fit, yellow = sum of peak fits, and light blue = residual. (c) Normalized and overlayed visible absorbance peaks from the peak fitting are shown for all of the polymers. (d) UV–vis spectrum of *p*-toluidine is shown.

The UV–vis spectra of the polymers were obtained to analyze the visible absorptions responsible for their colors (Figure 9a). A series of UV–vis spectra were obtained for each polymer at a range of concentrations (Figures S1–S11). All of the polymers adsorbed strongly in the UV region which overlapped with the peaks in the visible region. Therefore, peak fitting software was applied to predict ultraviolet and visible absorption maxima (see the Supporting Information). The UV region gave four absorptions consistent across all poly-NADs (**A**–**J**) (228–226, 291–303, 307–334, and 333–378 nm) (Figure 9b). To confirm that the color was not simply from the −NSS– linkage and involved the aromatic ring, the UV–vis spectrum of poly[*N*,*N*-(cyclohexylamine)disulfide] (**J**) was obtained (Figure S10). This polymer was colorless in solution and only had adsorptions in the UV region. From the UV–vis data for the polymers, it is clear that the ultraviolet region is complicated. All four absorptions in the UV region are present for **J** that overlap with the adsorptions for **A** through **I** that possess aromatic rings. The absorbance at ∼232 nm was attributed to the weak absorbance of the heterosubstituted disulfide linkage (−N–S–S–N−) consistent with previous reports.^36,57^ Substituents with non-bonding electrons demonstrated a bathochromic shift for all aromatic transitions with increasing π* stabilization. The visible absorptions also undergo a bathochromic shift and are hypothesized to be the n−π* transition of the nonbonding (−N–S−) to −N=S– transition stabilized by the aromatic ring and growing increasingly red-shifted with higher electron-donating substituents. This demonstrated that the polymers had significant conjugation through the backbone of the polymer, and electron-donating or -withdrawing substituents affected the visible adsorption. This is evidenced by the range of molar absorptivities with low values ranging from 62 to 278 L mol^–1^ cm^–1^ (Table 4). All visible absorption maxima were normalized to compare this bathochromic shift (Figure 9c). Notably, all starting monomers were white or pale yellow after purification and gave clear solutions until polymerized. An example of the UV–vis spectrum of one monomer—*p*-toluidine—is shown for comparison prior to polymerization (Figure 9d). The peaks for the E2 and B band aromatic π–π* transitions can be observed (300, 314 nm, respectively). Further studies need to be done to identify the origins of the peaks in the polymers.

**Table 4:** . Aggregate Visible Absorption Maxima and Calculated Molar Absorptivities of Poly-NADs (**A–I**)

poly-NAD	visible wavelength maxima (nm)	molar absorptivity (ε) at maxima (L mol^–1^ cm^–1^)
**A**	415.0	62.0
**B**	408.9	54.8
**C**	409.5	147.6
**D**	424.5	102.8
**E**	422.7	171.8
**F**	427.0	278.5
**G**	507.1	249.6
**H**	446.7	191.9
**I**	532.0	104.0

### 3.7 TGA of Selected Polymers

**Figure 10:**
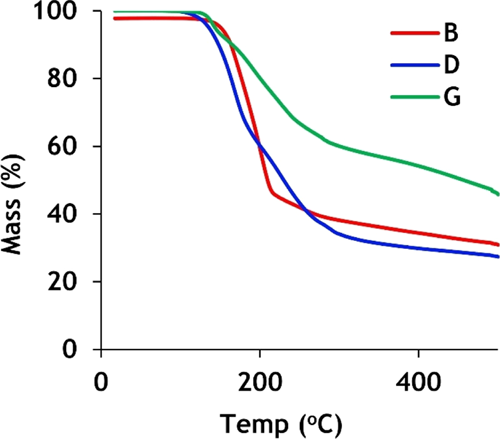
TGA spectra of polymer samples **B**, **D**, and **G** are shown.

TGA was performed to investigate the thermal decomposition of the selected examples (Figure 10). The TGA revealed the onset of decomposition at 170–180 °C for polymers **B**, **D**, and **G**. This result was similar to that found for a series of polymers synthesized from secondary diamines and a disulfide transfer reagent that possessed NSS functional groups.^37^ The disulfide bond is thermally labile in aliphatic bis-aminodisulfides. A qualitative measurement was conducted to observe changes in color at elevated temperatures by observing dissolved polymers (**A**-**I**) when heated to 80 °C for 2 h. The polymer colors were found to be unchanged (Figure S36).

### 3.8 Extending the Conjugation of the Polymer Side Groups

**Figure 11:**
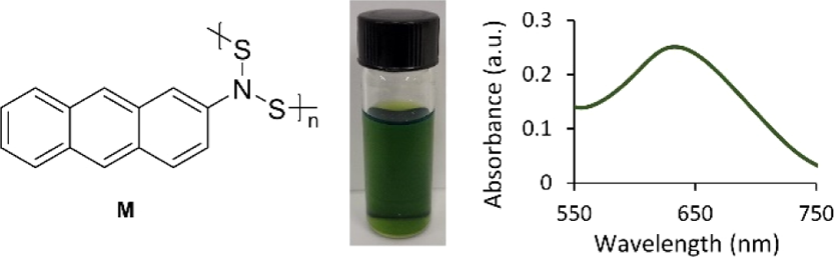
Green polymer synthesized from 2-aminoanthracene exhibited a shift in visible absorption with extended conjugation.

The polymerization of 2-aminoanthracene was completed under the same polymerization conditions as poly-NADs (**A–I**) to yield poly-[*N*,*N*-(2-aminoanthracene)disulfide] (**M**) with moderate molecular weight (Mw = 1600 g mol^–1^, *D̵* = 1.49) (Figure 11). This polymer demonstrates that by extending the conjugation of the side group we can elicit a more severe shift in the visible absorption to 635 nm to obtain a green color.

### 3.9 Sensors Based on Reactions of the NSS Backbone

The dependence of the aromatic ring for the color demonstrated conjugation between the aromatic ring and the NSS backbone. This conjugation led to a hypothesis that the reactivity of the backbone may differ for each polymer and that they could be used as colorimetric sensors. This hypothesis was investigated by exposing the polymers to 2-mercaptoethanol.

**Figure 12:**
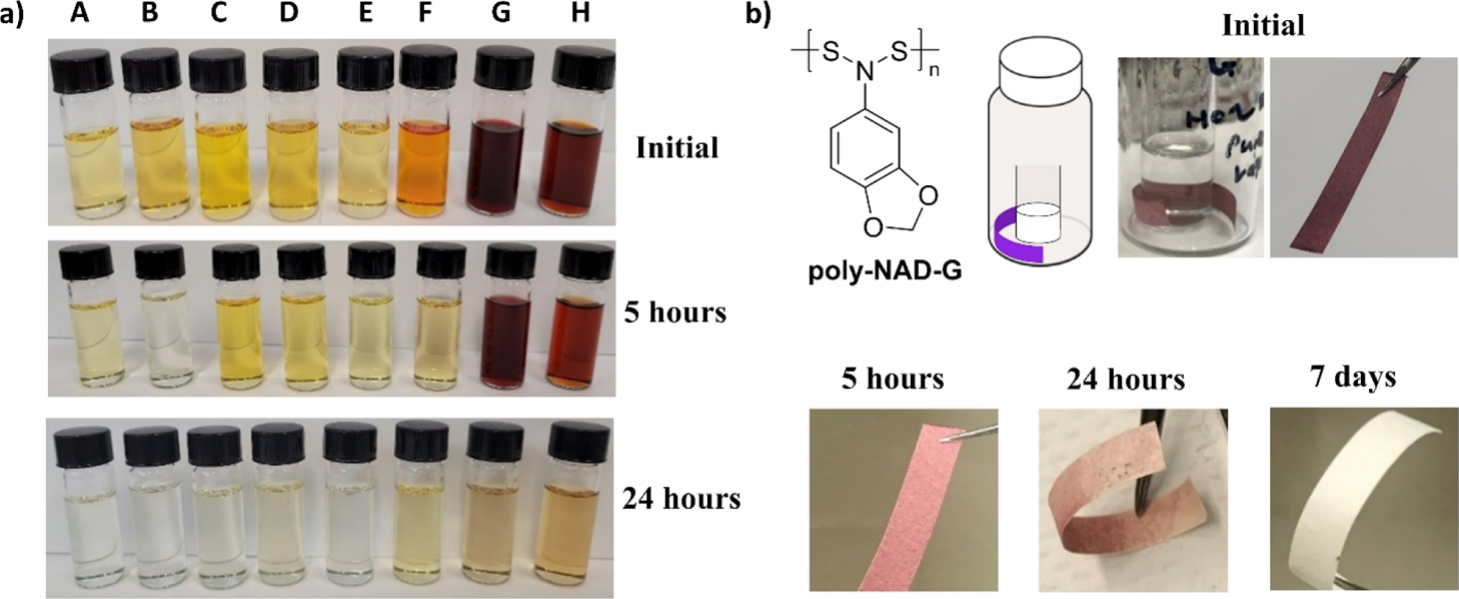
(a) Vials with solutions of poly-NADs (**A**–**H**, ∼3.0 mg/mL) were exposed to 2-mercaptoethanol (2-ME) (∼50 equiv, 0.5 mL) and the optical micrographs are shown. (b) Polymer **G** was coated on a polymer cellulose strip and exposed to vapors of 2-ME (2 mL). The color of the strips 5, 24 h, and 7 days after exposure to 2-ME are shown.

2-Mercaptoethanol was chosen because previous works demonstrated the sensitivity of heterosubstituted disulfides to thiols, and thiols have biological relevance.^58−60^ The polymers were dissolved in DCM at the same concentrations and then 50 equiv of 2-mercaptoethanol were added to each vial. Photographs of the vials before 2-mercaptoethanol was added and at 5 h and 24 h after its addition are shown in Figure 12a. At 24 h, all of the polymers had reacted with 2-mercaptoethanol and either yielded clear or slightly yellow vials. At 5 h, there was a difference in the extent of the reaction between the polymers and 2-mercaptoethanol. Some polymers, such as **B** and **F**, had reacted to yield clear or light yellow solutions. In contrast, polymers, such as **G** and **H**, were still strongly colored at 5 h. This result demonstrates that the polymers react at different rates and that these differences may be detected by monitoring their color. These are preliminary experiments; in future works, we will investigate how the molecular weight of the polymers as well as their composition affect their rates of the reaction and quantify these reactions using UV–vis spectroscopy.

In a second experiment, **G** was coated onto a cellulose-based filter paper by first dissolving it in DCM, dipping a paper strip into the solution, removing the paper, and allowing it to dry. The strip of paper was placed into a vial with a second open vial of 2-mercaptoethanol. The paper strip did not contact 2-mercaptoethanol liquid, but it was exposed to vapors of this chemical (bp = 157 °C). After 5 and 24 h, the color on the strip had noticeably changed and by a week, the strip was an off-yellow (Figure 12b). These preliminary experiments demonstrated that the polymer would react in the solid state and provides another potential route to use these polymers as colorimetric sensors. In future works, we will explore these applications.

## 4 Conclusions

In conclusion, a set of poly-NADs were synthesized by a one-step condensation polymerization and isolated in moderate yields. This new monomeric design provided a structurally unique backbone composed of one nitrogen and two sulfurs per monomeric unit and resulted in colorful polymers that suggest extended conjugation. In contrast to previous reports, these polymers contained both sulfurs bonded to the nitrogen rather than through the aromatic rings. Little to no reaction between sulfur monochloride and the aromatic rings were observed. The new structural motif of this set of polymers may provide interesting material applications from their possible electronic and photonic properties. Notably, these polymers were found to be air and moisture stable for extended periods of time, which contrasts the sensitivity of the phenylamine monomers to air oxidation. The polymers demonstrated interesting properties such as a unique array of colors and different levels of sensitivity to 2-mercaptoethanol. Future studies will investigate additional chemical sensitivities and physical/electrical properties of these polymeric materials.

## Supporting Information Available

UV–vis spectrum, Fityk peak-fitting of UV–vis spectrum and tabulated absorption maxima, and ^1^H NMR and ^13^C NMR spectroscopic data (PDF)
