# Supplementary material for: Polymerization of Aniline Derivatives to Yield Poly[N,N-(phenylamino)disulfides] as Polymeric Auxochromes
Source: Macromolecules. 2021 Nov 12;54(22):10405–14. doi: 10.1021/acs.macromol.1c01548 (PMC8619564; doi:10.1021/acs.macromol.1c01548)
Supplement: Supplementary file 1 — ma1c01548_si_001.pdf [file ma1c01548_si_001.pdf]

Supporting Information

Polymerization of Aniline Derivatives to Yield Poly[*N,N*-(phenylamino)disulfides] as Polymeric Auxochromes

James P. Grace, Evan S. Flitz, Dae Sun Hwang, Ned B. Bowden*

University of Iowa

Department of Chemistry

Iowa City, IA 52242

Ned-bowden@uiowa.edu

Table of Contents

UV-Vis Absorption Spectra of poly-NADs	S3-S6
Peak-fit UV-Vis Absorption Assignments	S6-S10
^{1}HNMR Spectroscopic Data	S11-S16
Qualitative Heat Sensitivity Experiment	S16
Elemental Analysis of Poly-NADs	S17
UV-Vis Absorption Spectra of Cross-linked Poly-NADs	S17

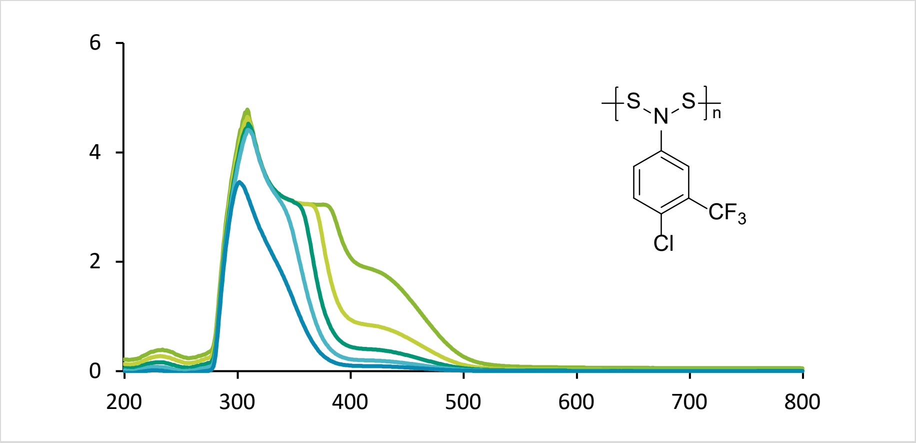

Figure S1. UV-vis spectrum of Poly-NAD **A** at five different concentrations performed by serial dilutions. Each spectra is half the concentration of the line above it.

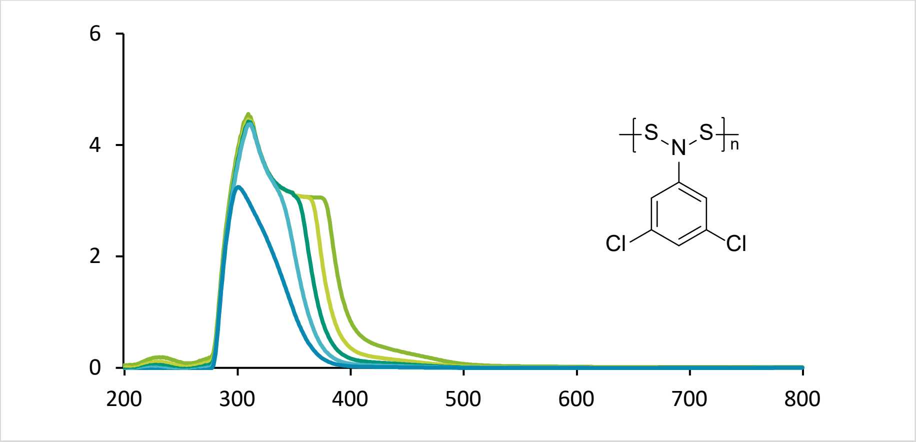

Figure S2. UV-vis spectrum of Poly-NAD **B** at five different concentrations performed by serial dilutions. Each spectra is half the concentration of the line above it.

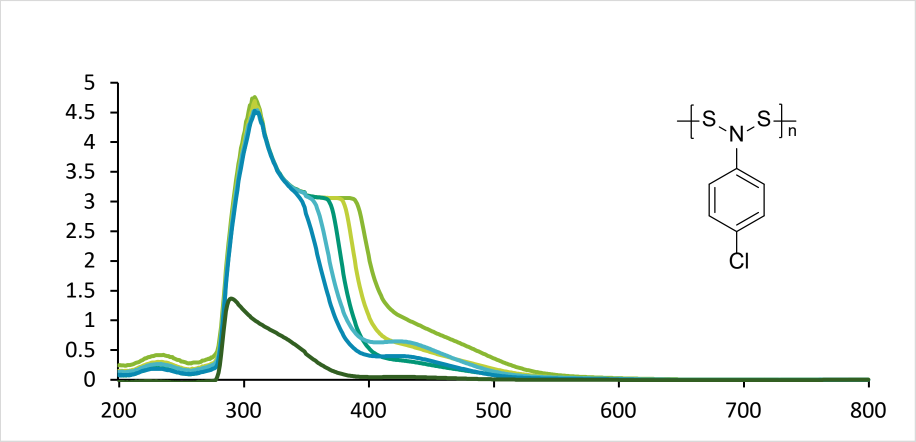

Figure S3. UV-vis spectrum of Poly-NAD **C** at six different concentrations performed by serial dilutions. Each spectra is half the concentration of the line above it except the final concentration which is 0.1x the concentration.

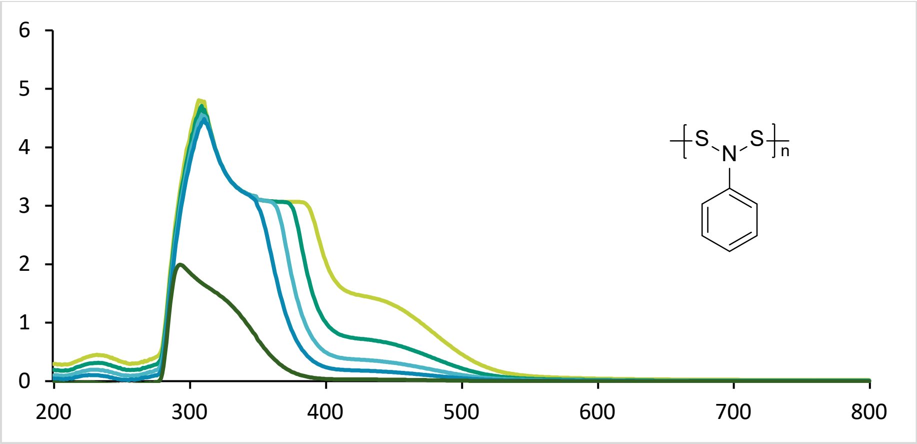

Figure S4. UV-vis spectrum of Poly-NAD **D** at five different concentrations performed by serial dilutions. Each spectra is half the concentration of the line above it.

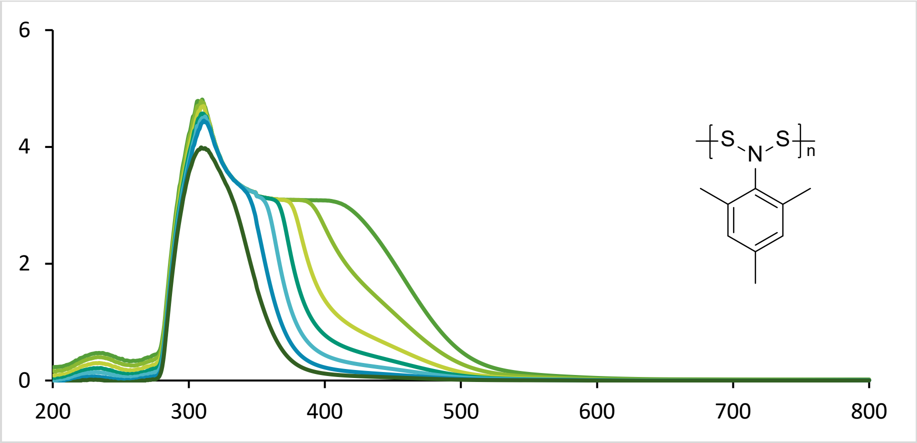

Figure S5. UV-vis spectrum of Poly-NAD **E** at seven different concentrations performed by serial dilutions. Each spectra is half the concentration of the line above it.

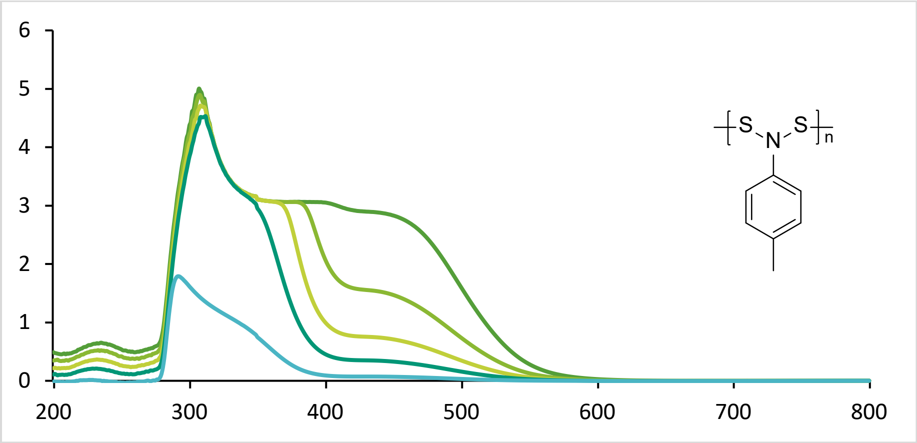

Figure S6. UV-vis spectrum of Poly-NAD **F** at five different concentrations performed by serial dilutions. Each spectra is half the concentration of the line above it.

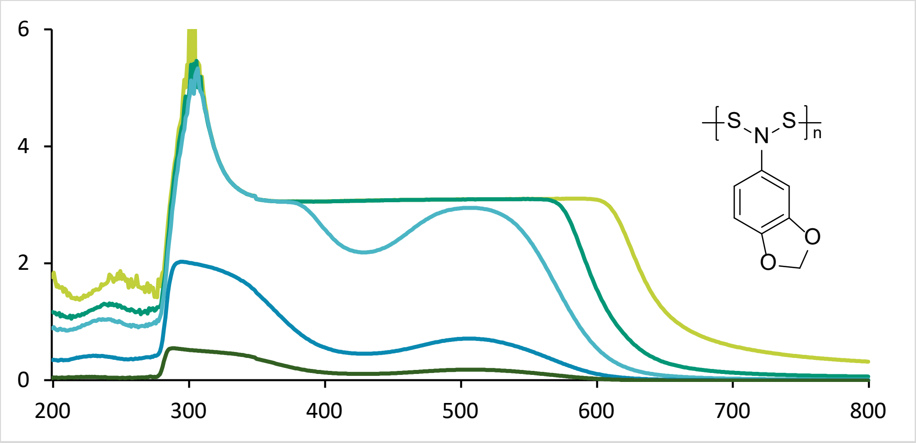

Figure S7. UV-vis spectrum of Poly-NAD **G** at five different concentrations performed by serial dilutions. Each spectra is half the concentration of the line above it.

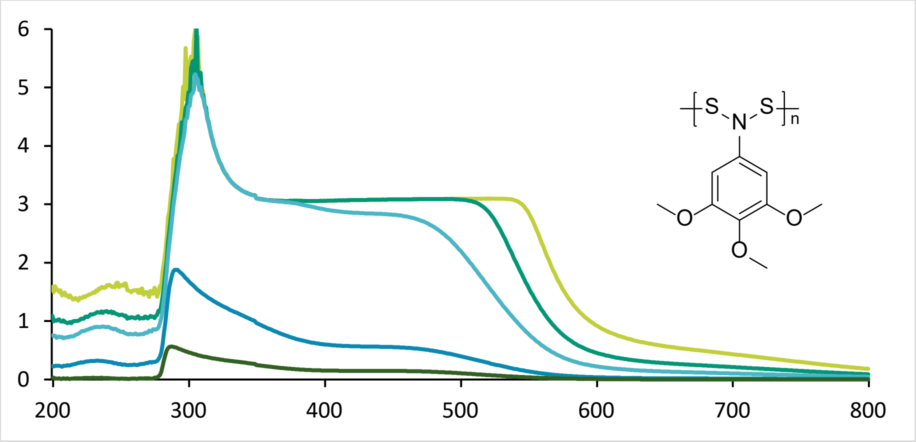

Figure S8. UV-vis spectrum of Poly-NAD **H** at five different concentrations performed by serial dilutions. Each spectra is half the concentration of the line above it.

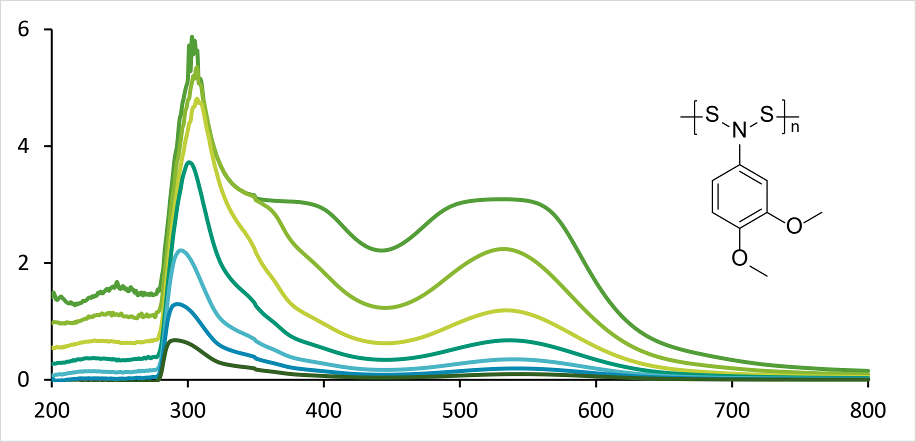

Figure S9. UV-vis spectrum of Poly-NAD **I** at seven different concentrations performed by serial dilutions. Each spectra is half the concentration of the line above it.

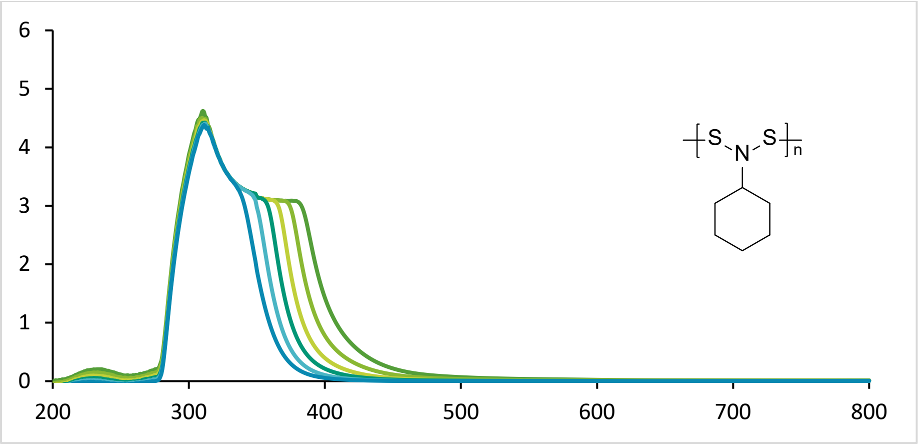

Figure S10. UV-vis spectrum of Poly-NAD **J** at six different concentrations performed by serial dilutions. Each spectra is half the concentration of the line above it.

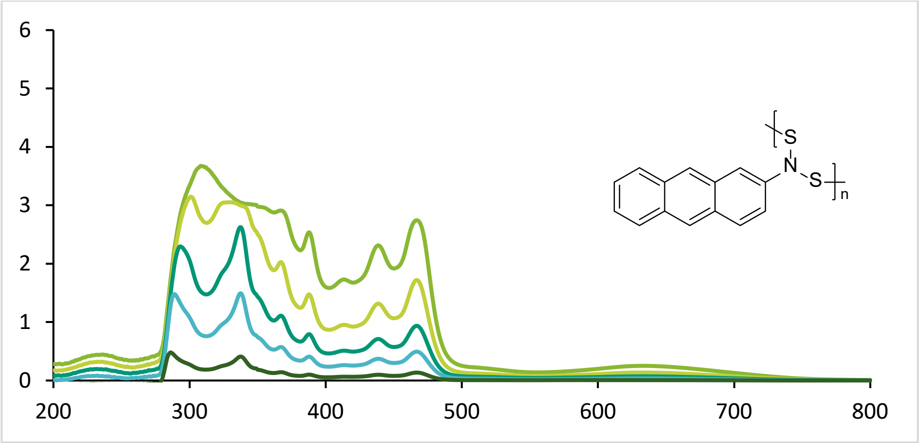

Figure S11. UV-vis spectrum of Poly-NAD **K** at five different concentrations performed by serial dilutions. Each spectra is half the concentration of the line above it.

<u>Peak Fitting of UV-Vis graphs</u>

The line colors in Figures S12 to S20 are as follows. dark blue line = parent graph, red lines = five peak-fit gaussian distributions, yellow line = summation of peak-fit gausian values, light blue line = residual value calculated by subtracting the summed peak-fit gaussian line from the parent graph.

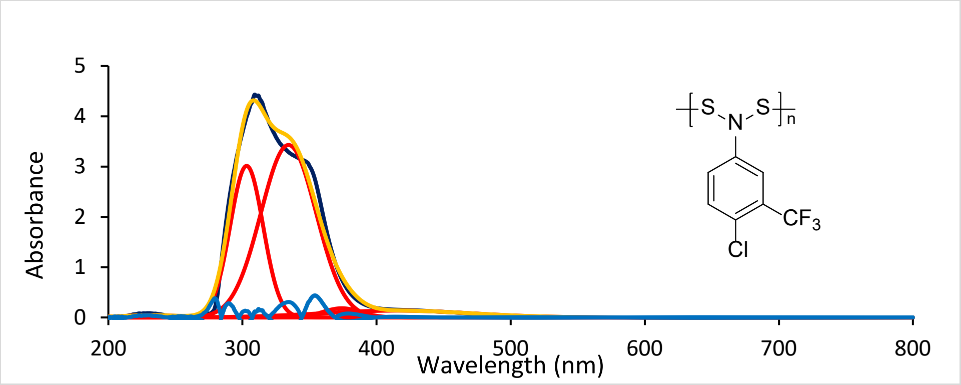

Figure S12. Peak-fit absorbances of UV-vis spectrum of Poly-NAD **A.**

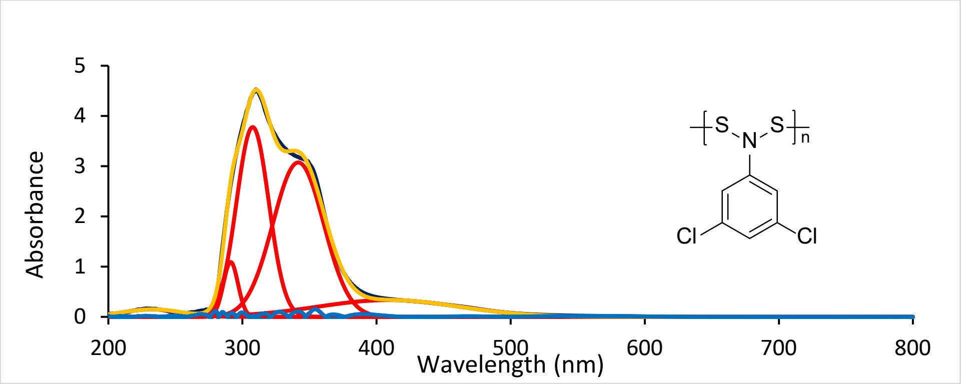

Figure S13. Peak-fit absorbances of UV-vis spectrum of Poly-NAD **B**

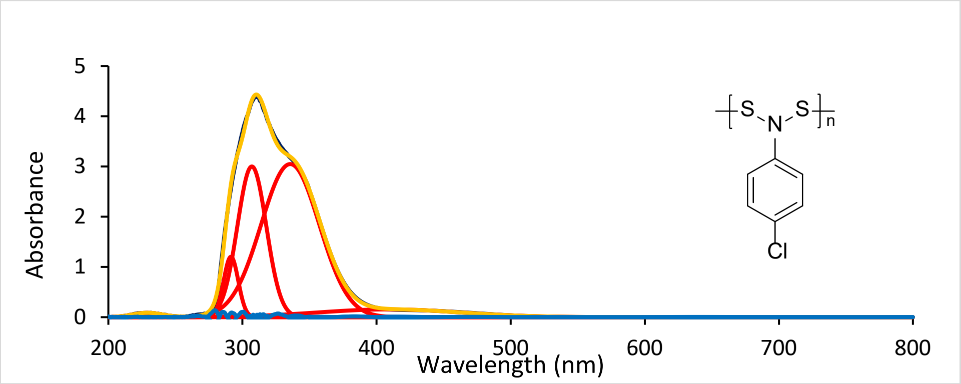

Figure S14. Peak-fit absorbances of UV-vis spectrum of Poly-NAD **C**

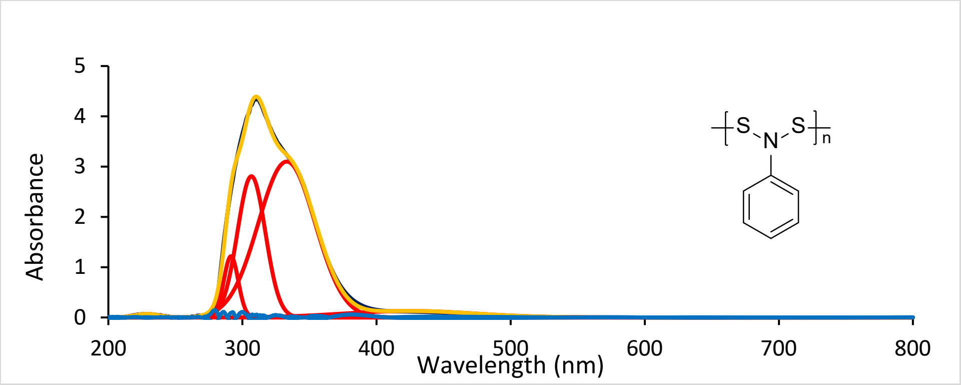

Figure S15. Peak-fit absorbances of UV-vis spectrum of Poly-NAD **D**

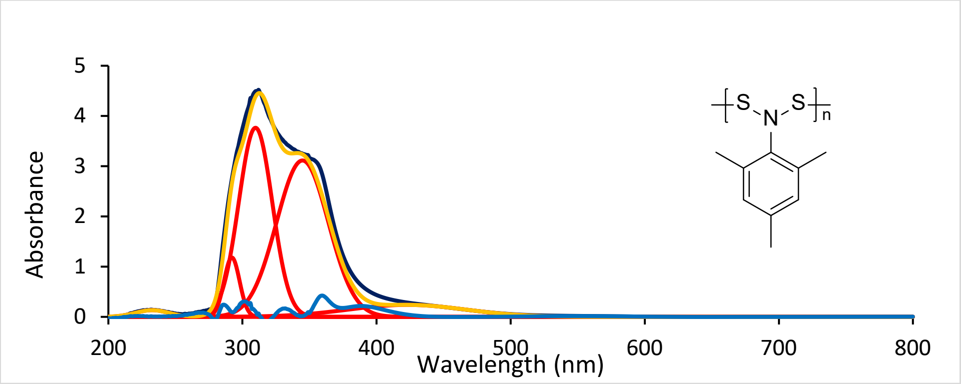

Figure S16. Peak-fit absorbances of UV-vis spectrum of Poly-NAD **E**

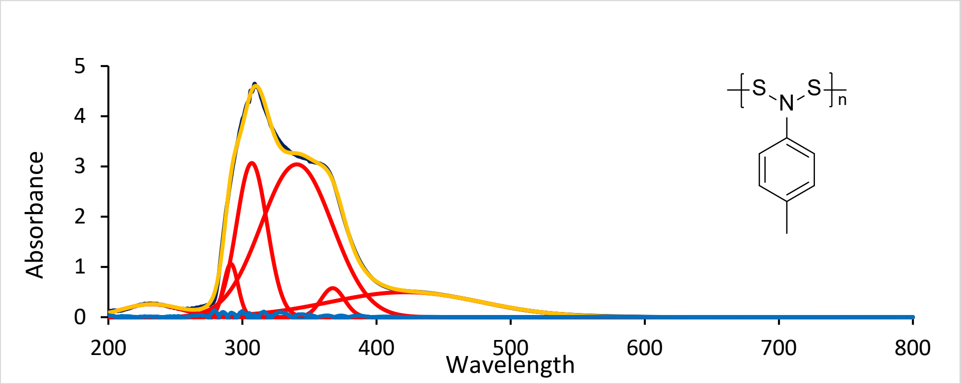

Figure S17. Peak-fit absorbances of UV-vis spectrum of Poly-NAD **F**

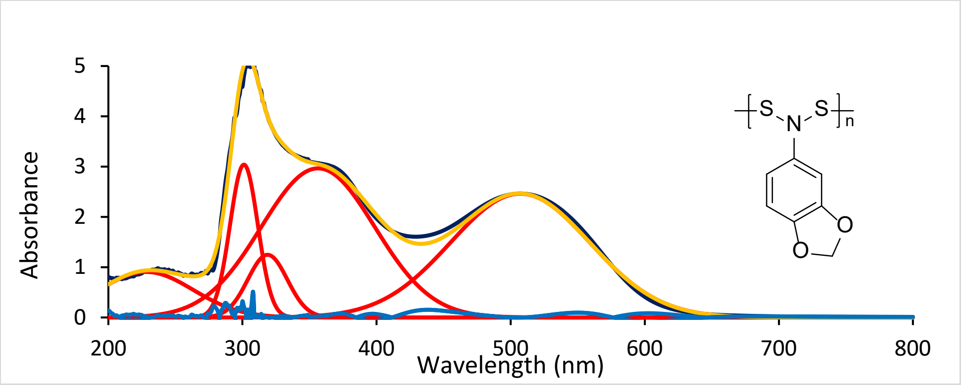

Figure S18. Peak-fit absorbances of UV-vis spectrum of Poly-NAD **G**

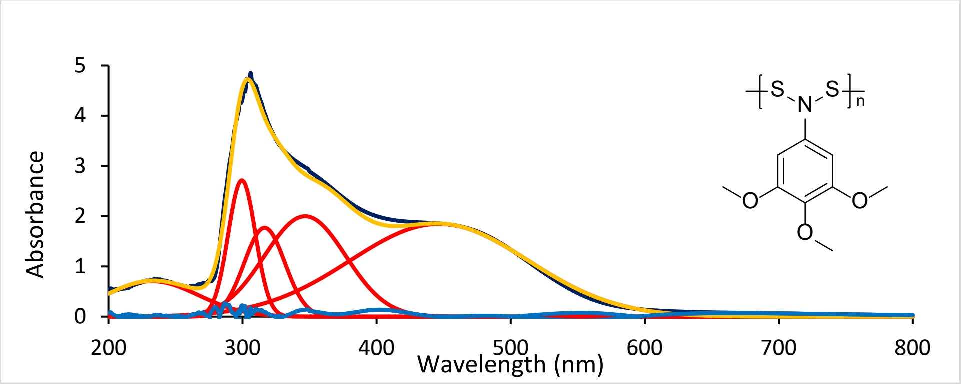

Figure S19. Peak-fit absorbances of UV-vis spectrum of Poly-NAD **H**

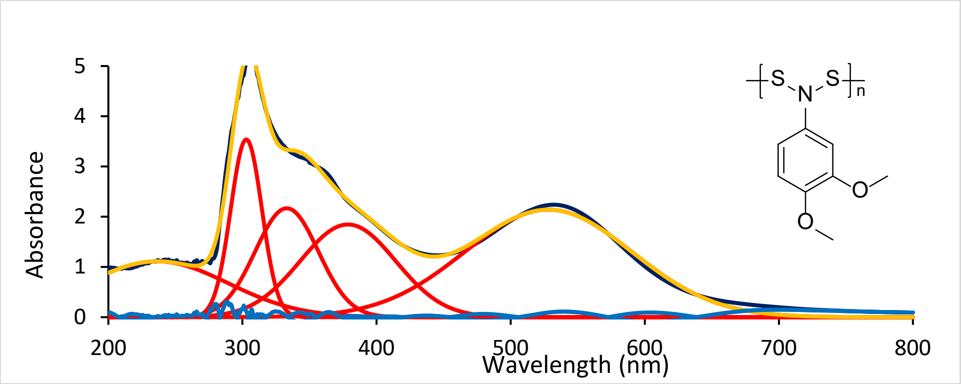

Figure S20. Peak-fit absorbances of UV-vis spectrum of Poly-NAD **I**

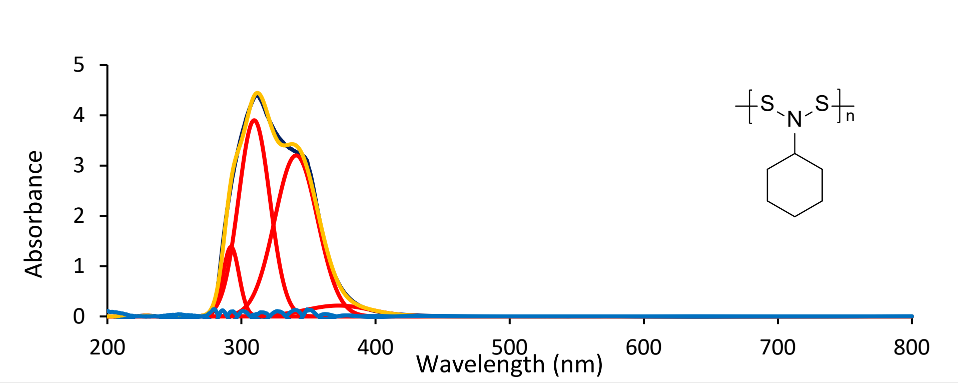

Figure S21. Peak-fit absorbances of UV-vis spectrum of Poly-NAD **J**

Table S1. Aggregate peak fit values of PolyNADs (A-I)

poly-NAD	Peak Fit 1	Peak Fit 2	Peak Fit 3	Peak Fit 4	Peak Fit 5
**A**	231.88	303.13	334.37	374.09	414.97
**B**	232.34	291.23	307.62	341.63	408.93
**C**	229.12	291.43	307.01	335.63	409.56
**D**	228.76	291.46	306.68	333.02	424.45
**E**	233.87	292.19	308.56	349.66	427.03
**F**	232.61	292.08	309.75	344.77	429.72
**G**	232.34	299.45	316.42	346.67	446.65
**H**	228.14	301.10	318.73	356.37	507.10
**I**	236.33	302.87	333.06	378.67	532.02
**Average**	231.71	296.10	315.80	351.16	N/A
**Standard Dev.**	1.99	4.72	8.89	12.16	N/A

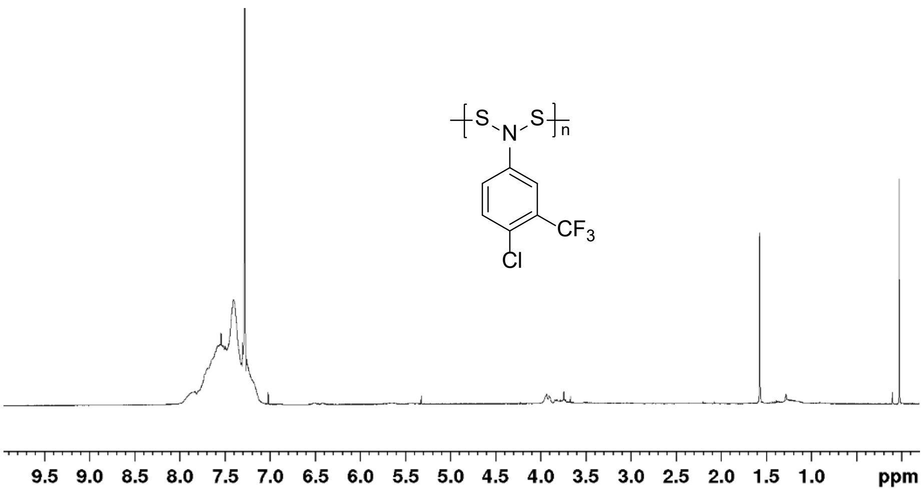

Figure S22. ^{1}H NMR spectrum of Poly-NAD (**A**)

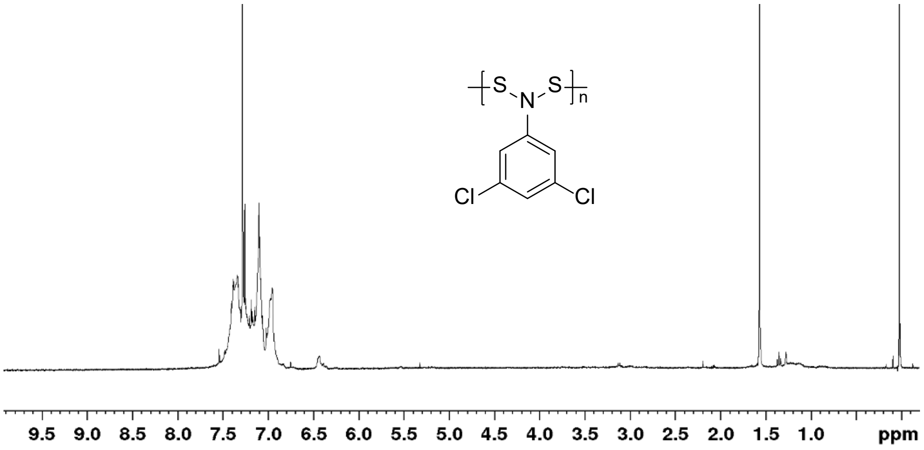

Figure S23. ^{1}H NMR spectrum of Poly-NAD (**B**)

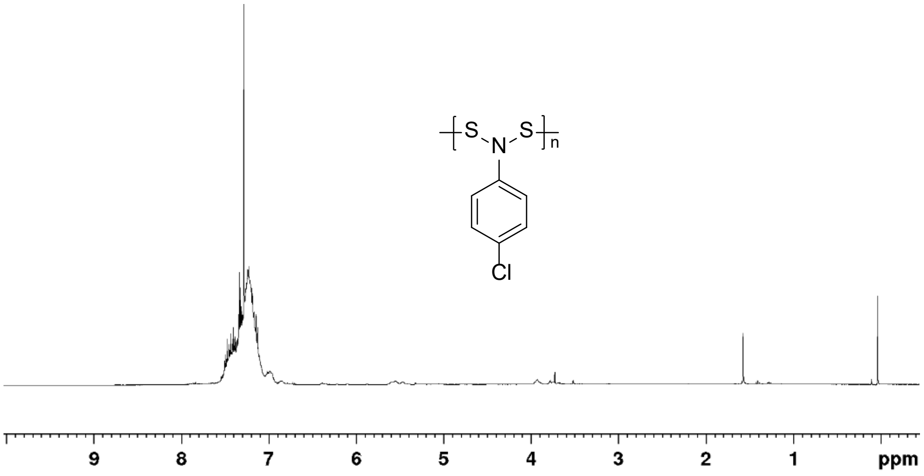

Figure S24. ^{1}H NMR spectrum of Poly-NAD (**C**)

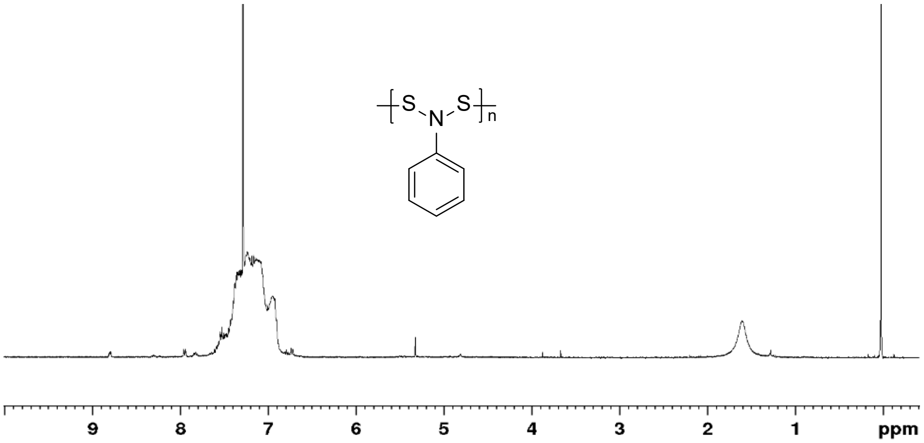

Figure S25. ^{1}H NMR spectrum of Poly-NAD (**D**)

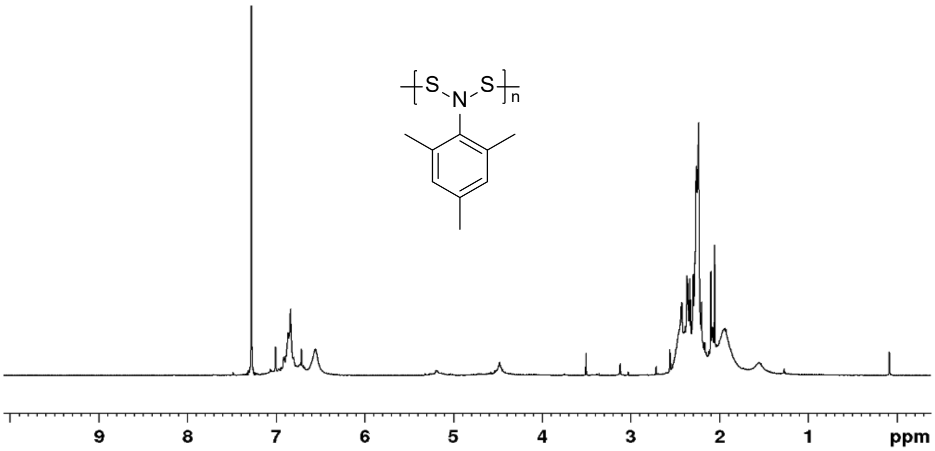

Figure S26. ^{1}H NMR spectrum of Poly-NAD (**E**)

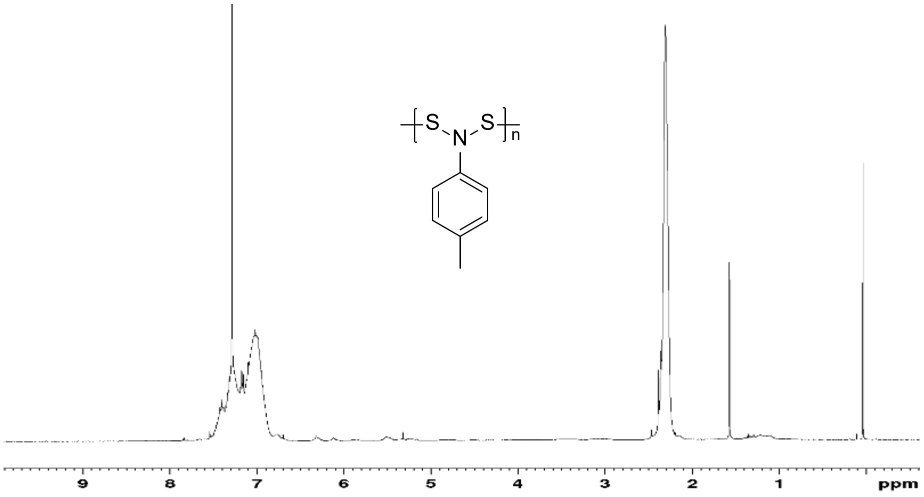

Figure S27. ^{1}H NMR spectrum of Poly-NAD (**F**)

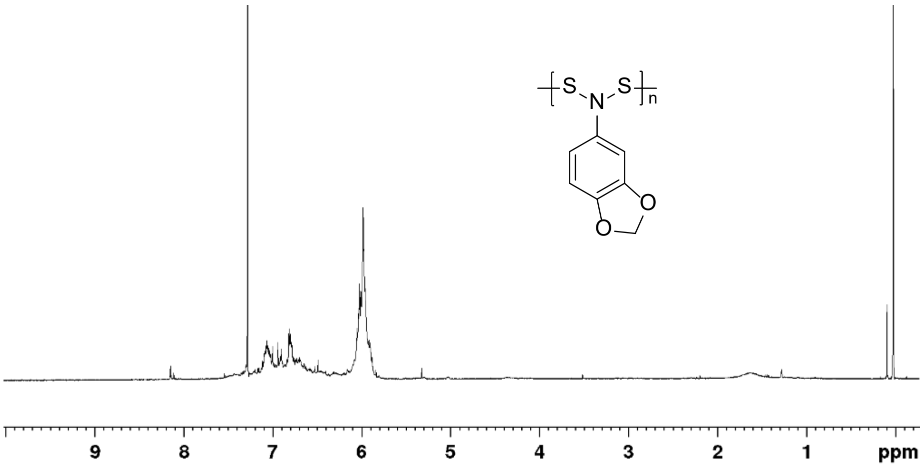

Figure S28. ^{1}H NMR spectrum of Poly-NAD (**G**)

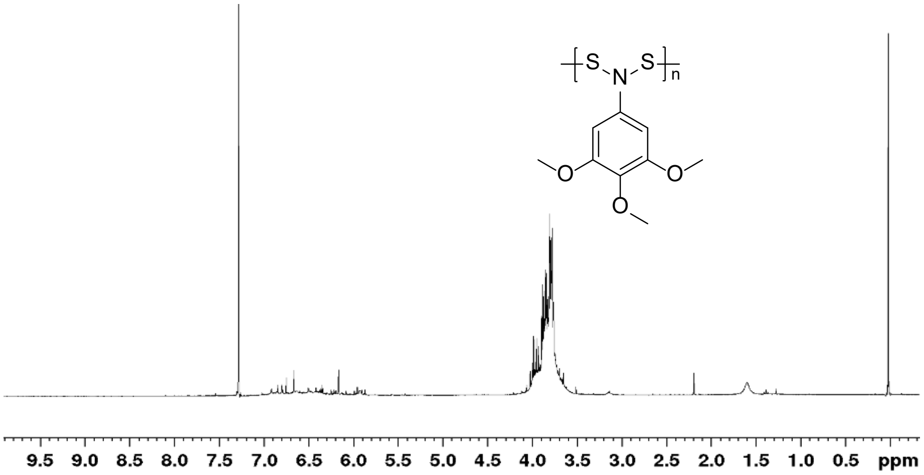

Figure S29. ^{1}H NMR spectrum of Poly-NAD (**H**)

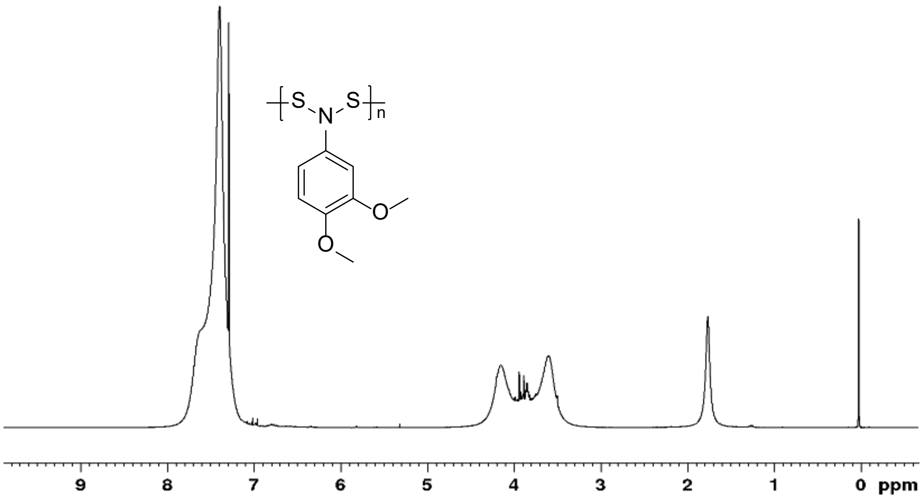

Figure S30. ^{1}H NMR spectrum of Poly-NAD (**I**)

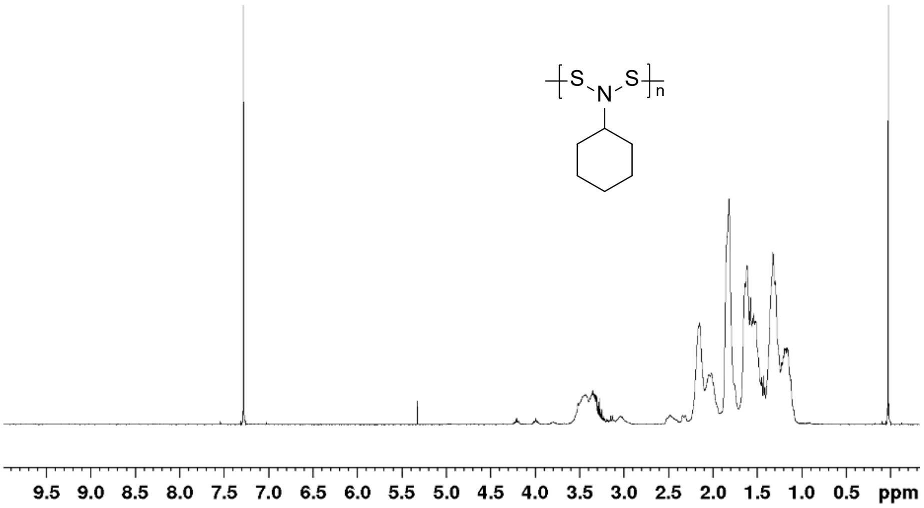

Figure S31. ^{1}H NMR spectrum of Poly-NAD (**J**)

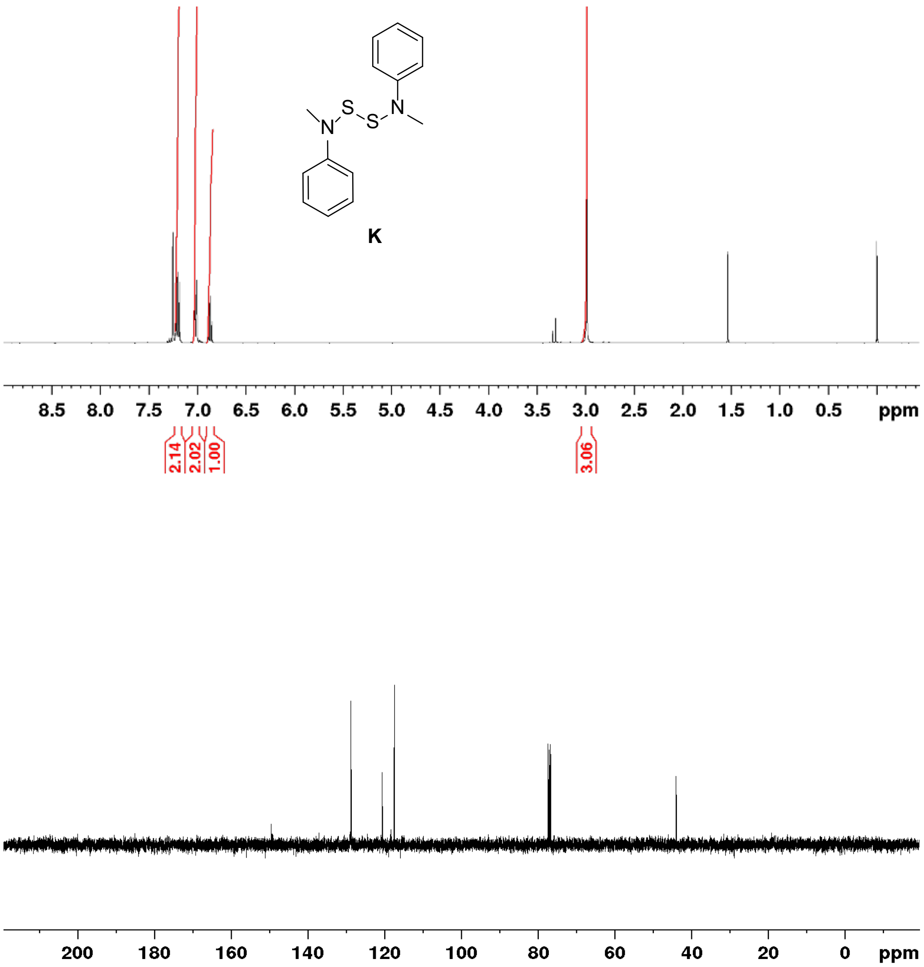

Figure S32. ^{1}H NMR spectrum and ^{13}C NMR spectrum of Bis-(*N*-methylaniline disulfide) (**K**)

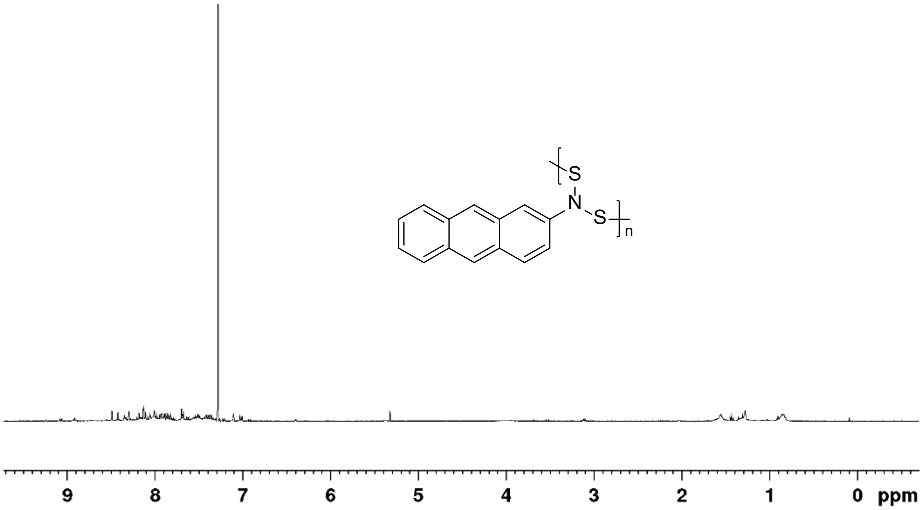

Figure S33. ^{1}H NMR spectrum of poly[*N,N*-(2-aminoanthracene)disulfide] (**M**)

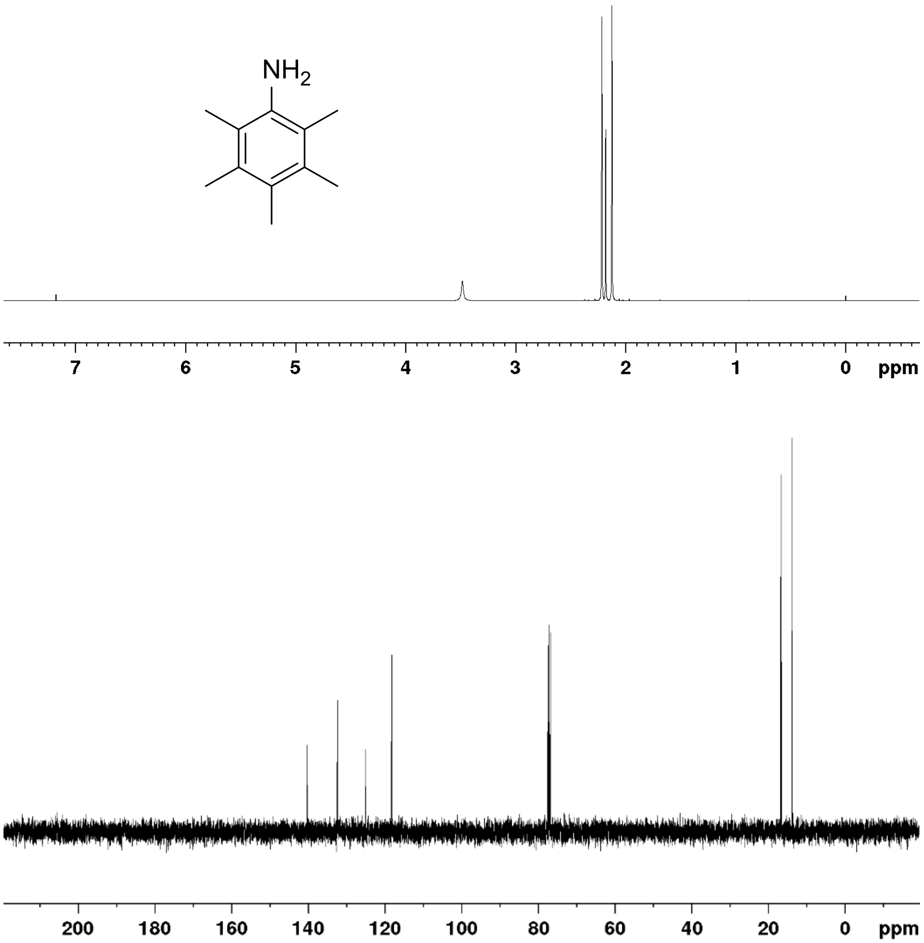

Figure S34. ^{1}H NMR spectrum and ^{13}C NMR spectrum of pentamethylaniline (**PMA**)

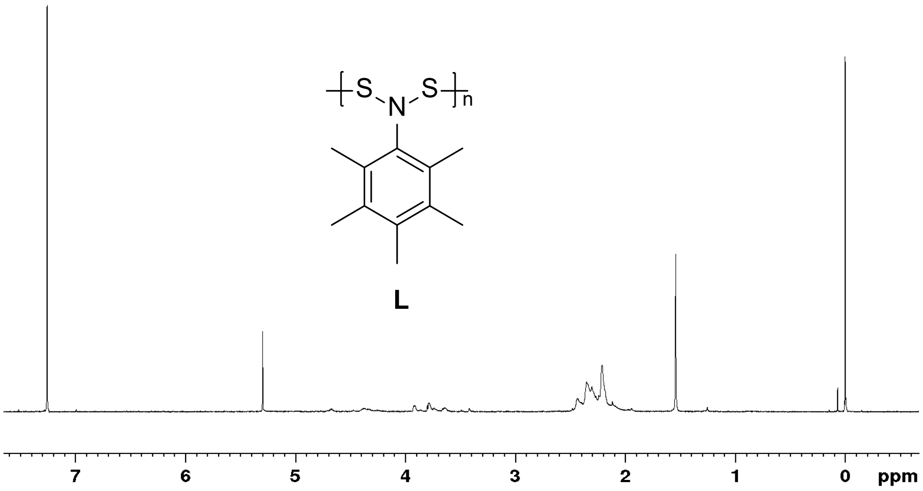

Figure S35. ^{1}H NMR spectrum of Poly-NAD (**L**)

Qualitative Heat Sensitivity Experiment

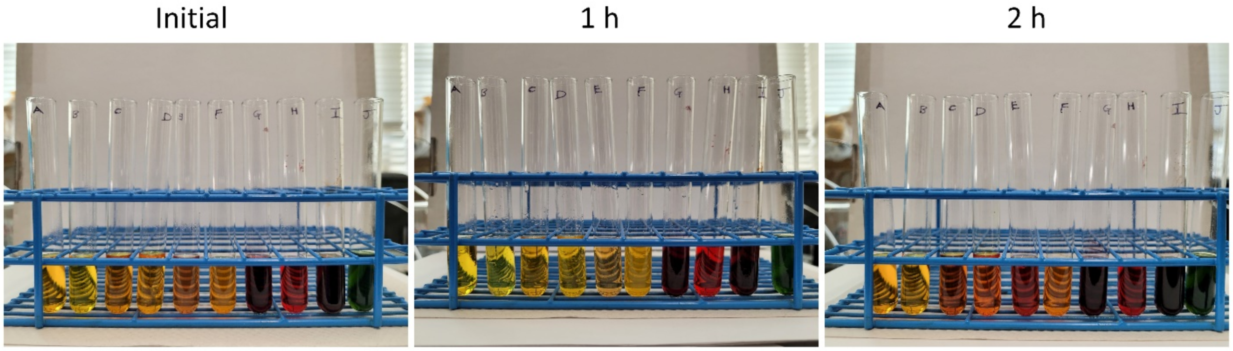

Figure S36. Poly-NADs A-I, J were dissolved at 6 mg/mL in toluene. They were heated to 80 ^{o}C and pictures were taken at 1 and 2 h. No change in color was observed.

Elemental Analysis of Poly-NADs (A-I)

Table S2. Elemental analysis of the composition of poly-NADs A-I reported as an average (n = 2).

	Polymer Sample Composition (%)				Calculated Monomer Composition (%)			
**Poly-NAD.**	**C**	**H**	**N**	**S**	**C**	**H**	**N**	**S**
**A**	30.4	1.0	5.1	30.5	32.6	1.2	5.4	24.9
**B**	31.0	1.3	6.0	31.1	32.2	1.4	6.3	28.6
**C**	31.4	1.8	6.1	45.7	38.0	2.1	7.4	33.8
**D**	43.8	3.0	8.5	47.0	46.4	3.3	9.0	41.3
**E**	45.9	4.9	6.0	41.6	54.8	5.6	7.1	32.5
**F**	44.5	4.1	7.9	42.4	49.7	4.2	8.3	37.8
**G**	38.5	2.2	6.2	36.8	42.2	2.5	7.0	32.1
**H**	40.1	2.4	6.7	33.0	44.1	4.5	5.7	26.1
**I**	38.6	3.8	5.7	36.9	44.6	4.2	6.5	29.8

UV-Vis of crosslinked Poly-NADs with 5% p-pda

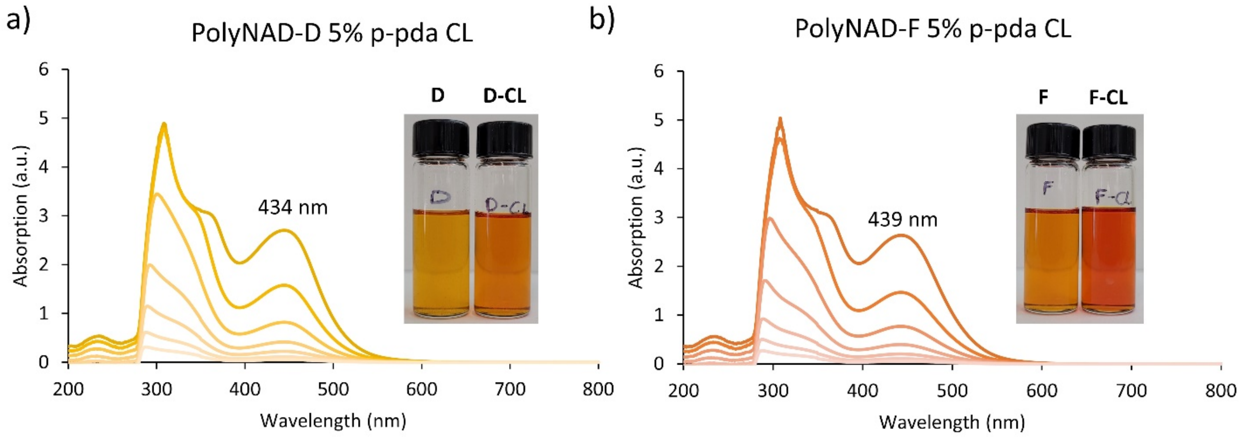

Figure S37. UV-vis spectrum of a) cross-linked Poly-NAD **D** with 5% ppda and b) cross-linked Poly-NAD **F** with 5% ppda. Each spectra was recorded at seven different concentrations performed by serial dilutions. Each spectra is half the concentration of the line above it. The pictures show the qualitative measurement of the polymers at the same concentration (2 mg mL^{-1}) in DCM compared to the uncrosslinked analogues.
